# Biocontrol Potential of a Novel Endophytic Bacterium From Mulberry (*Morus*) Tree

**DOI:** 10.3389/fbioe.2019.00488

**Published:** 2020-01-23

**Authors:** Sen Xie, Marine Vallet, Chao Sun, Maritta Kunert, Anja David, Xiancui Zhang, Bosheng Chen, Xingmeng Lu, Wilhelm Boland, Yongqi Shao

**Affiliations:** ^1^Institute of Sericulture and Apiculture, College of Animal Sciences, Zhejiang University, Hangzhou, China; ^2^Max Planck Fellow Group on Plankton Community Interaction, Max Planck Institute for Chemical Ecology, Jena, Germany; ^3^Analysis Center of Agrobiology and Environmental Sciences, Zhejiang University, Hangzhou, China; ^4^Department of Natural Product Biosynthesis, Max Planck Institute for Chemical Ecology, Jena, Germany; ^5^Department of Bioorganic Chemistry, Max Planck Institute for Chemical Ecology, Jena, Germany; ^6^Key Laboratory for Molecular Animal Nutrition, Ministry of Education, Hangzhou, China

**Keywords:** endophyte, fungal pathogen, lepidopteran pest, mulberry, symbiosis

## Abstract

Mulberry (*Morus*) is an economically important woody tree that is suitable for use in sericulture as forage and in medicine. However, this broad-leaved tree is facing multiple threats ranging from phytopathogens to insect pests. Here, a Gram-positive, endospore-forming bacterium (ZJU1) was frequently isolated from healthy mulberry plants by screening for foliar endophytes showing antagonism against pathogens and pests. Whole-genome sequencing and annotation resulted in a genome size of 4.06 Mb and classified the bacterium as a novel strain of *Bacillus amyloliquefaciens* that has rarely been identified from tree leaves. An integrative approach combining traditional natural product chemistry, activity bioassays, and high-resolution mass spectrometry confirmed that strain ZJU1 uses a blend of antimicrobials including peptides and volatile organic compounds to oppose *Botrytis cinerea*, a major phytopathogenic fungus causing mulberry gray mold disease. We showed that the inoculation of endophyte-free plants with ZJU1 significantly decreased both leaf necrosis and mortality under field conditions. In addition to the direct interactions of endophytes with foliar pathogens, *in planta* studies suggested that the inoculation of endophytes also induced plant systemic defense, including high expression levels of mulberry disease resistance genes. Moreover, when applied to the generalist herbivore *Spodoptera litura*, ZJU1 was sufficient to reduce the pest survival rate below 50%. A previously undiscovered crystal toxin (Cry10Aa) could contribute to this insecticidal effect against notorious lepidopteran pests. These unique traits clearly demonstrate that *B. amyloliquefaciens* ZJU1 is promising for the development of successful strategies for biocontrol applications. The search for new plant-beneficial microbes and engineering microbiomes is therefore of great significance for sustainably improving plant performance.

## Introduction

Microorganisms colonizing plant surfaces (the rhizosphere and phyllosphere) and inner tissues (the endosphere) play an important role in plant host health and productivity (Barra et al., 2018; Cordovez et al., 2019). Recent high-throughput approaches targeting the entire microbiota, such as whole-metagenome sequencing, have shown that bacteria dominate all plant compartments and perform various beneficial activities (Remus-Emsermann et al., 2012; Leach et al., 2017). For instance, common phyllosphere bacteria belonging to the genus *Sphingomonas* protect *Arabidopsis* against phytopathogens by reducing pathogen growth (Innerebner et al., 2011). Furthermore, the transplantation of rhizosphere bacterial communities from resistant tomato suppresses disease symptoms in susceptible cultivars (Kwak et al., 2018), demonstrating the potential for microbiome engineering to increase crop yield (Foo et al., 2017; Dini-Andreote and Raaijmakers, 2018).

In contrast to well-studied phyllosphere and rhizosphere bacteria, only a few studies have specifically examined foliar endophytic bacteria (Ding et al., 2013; Müller et al., 2015). In general, foliar bacterial endophytes occur at low population densities inside plants; however, they can establish a mutualistic relationship with their hosts (Rosenblueth and Martínez-Romero, 2006). For instance, several endophytic *Bacillus* species produce highly diverse antimicrobials against a variety of phytopathogens, insects and nematodes, indicating potential application in biocontrol (Lopes et al., 2018). Therefore, a more thorough examination of endophytes in diverse plants may result in numerous additional microbial resources that may be used in bioengineering and biotechnology to improve agricultural productivity.

Mulberry (*Morus* spp.) is a woody perennial tree that is widely cultivated throughout subtropical and temperate regions (Berg, 2001). Mulberry is well-known as an economically important feed crop for the domesticated silkworm (*Bombyx mori*) in sericulture but also attracts farmers because of its delicious fruit and multiple uses in traditional medicine (He et al., 2013). However, mulberry plants are unfortunately facing multiple threats from phytopathogens and herbivores. For instance, gray mold caused by the fungal pathogen *Botrytis cinerea* is a widespread and destructive disease of mulberry trees (Elad et al., 2004). Necrotrophic *B. cinerea* is difficult to control or prevent because it exhibits multifarious modes of attack and a wide host range (Droby and Lichter, 2007), and it can persist as mycelia and/or conidia for extended periods as sclerotia in plant debris (Williamson et al., 2007). In particular, *B. cinerea* has already developed multiple resistance to several or even all currently used fungicides, which is probably promoted by their excessive use (Weber and Hahn, 2019). Moreover, mulberry is often attacked by a number of insect pests, mostly belonging to the Lepidoptera (Chen B. et al., 2018; Chen et al., 2019). Since mulberry leaves are used to feed silkworms or are processed into pharmaceutical products, the improper use of agrochemicals to treat these diseases and herbivores could be hazardous to silkworms and even humans. Biocontrol appears to offer a valid alternative to solve this problem. An endophytic bacterium, *Burkholderia cepacia*, that has previously been isolated from mulberry leaves, inhibits the plant-pathogenic fungus *Colletotrichum dematium* (Ji et al., 2010). However, *B. cepacia* is an opportunistic human pathogen and attacks young plants such as tobacco, limiting its possible use as a biocontrol agent (Shommu et al., 2015). Considering that diverse bacteria may live inside a leaf, the investigation of new endophytes from mulberry plants has become essential.

In the present study, we screened leaf endophytes associated with healthy trees in a mulberry-planting field and identified the Gram-positive, endospore-forming bacterial strain *Bacillus amyloliquefaciens* ZJU1, showing antagonism against both *B. cinerea* and the generalist insect herbivore *Spodoptera litura* (Chen et al., 2016b). Endophytic *B. amyloliquefaciens* has been isolated from the seeds and stems of grass plants such as rice (*Oryza sativa*) and the herb *Bacopa monnieri* and is known to provide benefits to host plants (Lopes et al., 2018). However, *B. amyloliquefaciens* has rarely been isolated from the leaves of woody plants. Thus, *B. amyloliquefaciens* ZJU1 represents a novel strain for examining the potential of biocontrol and other bioapplications. Currently, whole-genome sequencing is poised to provide substantial information for understanding key bacterial characteristics (Liang et al., 2018). Together with sequencing efforts, comparative genomic analysis of this strain with other strains isolated from various environments provided further insight into the pan- and core genomes, suggesting the existence of functional diversity in *Bacillus* species. Based on both genetic and chemical characterization, we aimed to elucidate the possible mechanisms of this antagonism to lay a foundation for future biocontrol applications.

## Materials and Methods

### Sample Collection and DNA Extraction

Five healthy mulberry trees were randomly selected from a mulberry-planting field in Hangzhou, China (30°18′6.31″ N, 120°05′9.25″ E), and leaves were collected randomly from different branches of each sampled tree in October 2017. For sampling, surface-disinfected gloves and razor blades as well as sterile bags were used. The individual leaves were severed aseptically from the petioles by using a sterile razor blade, placed a sterile bag, and kept on ice until further processing. The collected leaves were washed with sterile water to remove dust and other debris before being placed in 0.25% NaOCl and 80% EtOH (3 min each step) to remove leaf surface microorganisms. The samples were then rinsed three times in sterile water (1 min each time) again and dried on sterile filter paper to remove excess water. To assess the efficacy of surface disinfection, 100 μL of the last rinse was plated on a PDA plate and incubated at 30°C for 4 days. The plates were examined for the absence or presence of microorganism colony growth. Leaf samples that showed any microbial growth from the last rinse water (the control) were removed from further analyses. The endophytic bacteria were isolated from the surface-sterilized mulberry leaves using the fragmentation technique (Liotti et al., 2018) as follows: the cleaned leaves were aseptically cut into 1 × 1 cm pieces, placed on PDA plates and incubated at 30°C for 4 days. The resulting bacterial colonies were randomly picked and subcultured at least three times before identification. The genomic DNA of pure cultures was extracted using the MasterPure™ Complete DNA and RNA Purification Kit (Epicenter, Madison, USA) according to the manufacturer's instructions.

### Genome Sequencing, Assembly and Annotation

The whole genome of *B. amyloliquefaciens* ZJU1 was sequenced using PacBio RSII in Majorbio Bio-pharm Technology Co., Ltd. (Shanghai, China). 1,154 Mbp high-quality reads were generated, and filtered by following 5′ end containing non-A, G, C, and T bases, sequences shorter than 25 bps, sequences contained with 10% “N” bases, adaptor sequences and low quality reads with quality scores <20. SOAPdenovo V1.05 was used to generate contigs and scaffolds using k-mer sizes and gaps were filled closed by PCR amplification method (Chen et al., 2016a; Jia et al., 2017). The raw dataset, including both single and paired end reads (average read length of 7,483 bp) is deposited at DDBJ/EMBL/GenBank under BioProject ID: PRJNA544619, SRR9125050. Genes of strain ZJU1 were predicted by Glimmer V3.02 software (http://www.cbcb.umd.edu/software/glimmer/). The predicted protein sequences were compared against the Nr, genes, string, and GO databases using BLAST 2.2.28+ and a cut off value 1e^−5^ (Altschul et al., 1990; Ashburner et al., 2000; Li et al., 2002), respectively. To obtain annotation information, sequences were further compared against the Clusters of Orthologous Group sequence database (COG, http://www.ncbi.nlm.nih.gov/COG/) and Kyoto Encyclopedia of Genes and Genomes database (KEGG, https://www.genome.jp/kegg/) using cutoff value 1e^−5^. GI island sequences were predicted by three methods: IslandPATH-DIMOB, SIGI-HMM, and IslandPick, and visualized on the chromosome. Transfer RNA (tRNA) and ribosome RNA (rRNA) genes were detected by tRNAscan-SE V1.3.1 (http://lowelab.ucsc.edu/tRNAscan-SE/index.html) and Barrnap V0.4.2 (http://www.vicbioinformatics.com/software.barrnap.shtml), respectively (Lowe and Eddy, 1997). Interspersed repetitive sequences were identified from genomic sequences using RepeatMasker (http://www.repeatmasker.org), and tandem repeats using Tandem Repeats Finder (TRF) (Benson, 1999; Saha et al., 2008). Prediction of prophage was performed by PHAST (http://phast.wishartlab.com) (Zhou et al., 2011). Furthermore, CRISPRFinder online tool (http://crispr.i2bc.paris-saclay.fr/) was used for CRISPR identification (Grissa et al., 2007). Genomic circle map was visualized by Circos V0.64 (http://circos.ca/).

### Multi-Genome Analysis

A range of complete genome sequences with protein functions of genus *Bacillus* were downloaded from NCBI database (12th March 2019, https://www.ncbi.nlm.nih.gov/genome/). MUMER V3.0 was applied for identification of homology regions and for collinearity analysis by default parameters (Kurtz et al., 2004). The inferred collinear genes were used for further phylogenetic and evolutionary analyses. Pan-genome analysis was performed using PGAP, and changes in the number of core genes were predicted by fitting the exponential decay function: ${\text{F}}_{\text{e}}\left(\text{n}\right)={{\text{k}}_{\text{c}}\text{e}}^{-n/\tau_{c}}+\Omega$. Meanwhile, the pan-genome size was predicted by power function fitting: F(n) = kn^γ^. When γ < 0, it is considered to be a closed pan-genome, and γ ≥ 0, an open pan-genome. *Bacillus* genomes were selected for homologous gene analysis by OrthoMCL (http://orthomcl.org/common/downloads/software/v2.0/) with thresholds as follows: E-Value, 1e-5; Markov Inflation Index, 1.5. Based on homologous gene clustering analysis, single copy homologous genes were selected for multiple sequence alignment by MAFFT (https://mafft.cbrc.jp/alignment/software/), and Gblocks (http://molevol.cmima.csic.es/castresana/Gblocks.html) was used for quality control. Phylogenetic tree based on single copy genes was constructed using RAxML (https://github.com/stamatak/standard-RAxML). antiSMASH was employed to identify secondary metabolite gene clusters in the ZJU1 genome (Blin et al., 2019).

### Antagonism Test Against the Pathogen *B. cinerea* and the Insect Pest *S. litura*

To test the biocontrol ability of strain ZJU1 against pathogenic fungi, ZJU1 and *B. cinerea* cultures were inoculated simultaneously on PDA plates. *Bacillus cereus* was used as a negative control. Furthermore, a fresh ZJU1 culture was inoculated into PDB medium (200 g/L potato, 20 g/L glucose, pH 5.6 ± 0.2) and grown at 30°C. The bioassay was performed according to previous studies (Shao et al., 2017). Briefly, a 1 mL aliquot of a culture collected after 24 h of incubation was centrifuged at 13,000 rpm for 5 min to collect the supernatant. A 50 μL aliquot of the resulting supernatant was used in the well diffusion assay to observe antagonistic activity against *B. cinerea*. For *in planta* infestation experiments, fresh mulberry leaves were inoculated with strain ZJU1, the negative control *B. cereus* and the challenge pathogen *B. cinerea* (mycelial plug excised from a PDA plate) and then covered with a polythene bag for 48 h to maintain humidity and avoid any disturbance. Disease incidence and lesion sizes were surveyed. Subsequently, the inoculated leaves were excised and immersed in 80% acetone to extract the chlorophyll contents as previously described (Ritchie, 2006). The absorbance was measured at wavelengths of 645 and 663 nm, respectively. Total chlorophyll content was determined by the following equations: total chlorophyll (μg/mg) = 8.02 (A_663_) + 20.2 (A_645_) (Arnon, 1949).

Total RNA from the infested leaves was extracted using the MasterPure™ Complete DNA and RNA Purification Kit (Epicenter, Madison, USA) to investigate the expression of plant disease resistance genes. The extracted RNA was further reverse transcribed to cDNA using random primers (Vazyme Biotech, Nanjing, China). Quantitative RT-PCR (qRT-PCR) was carried out in a Roche LightCycler 480 system (Roche, Basel, Switzerland) using SYBR® qPCR master mix (Vazyme Biotech, Nanjing, China). The primer sequences for all test genes are shown in Table S1. RT-PCR was conducted using the following program: 5 min of denaturation at 95°C, followed by 40 cycles of 10 s at 95°C, 10 s of annealing at an appropriate temperature, and 10 s of elongation at 72°C, followed by a final melting curve step (from 65 to 92°C, 0.5°C/s). Each sample included at least five replicates.

To investigate the insecticidal properties of strain ZJU1, different bacterial cultures were prepared and mixed with artificial feed (pinto bean-based diets) at a dose of 7 x 10^3^ CFU per gram of feed. *S. litura* larvae were maintained under three regimes: (i) no treatment, (ii) feed containing strain ZJU1, and (iii) feed containing *B. cereus* as a negative control. The diet was changed every 24 h. The disease symptoms and survival rates were recorded until pupation.

For protein structure prediction, the Cry10Aa sequences (Gene ID: 9779107; 5759939) were searched against BlastN and further analyzed by Geneious V5.5.7 (Kearse et al., 2012). The resulting amino acid sequences were aligned with the CLUSTALW MUSCLE algorithm. The secondary and tertiary structures of Cry10Aa were predicted by Phyre2 using structure templates and default parameters extracted from the Protein Data Bank (PDB) (Kelley and Sternberg, 2009).

### *In situ* Metabolite Extraction, UHPLC-HR-MS and GC-MS Analyses

For the cocultivation of the endophyte bacterium ZJU1 and the pathogenic fungus *B. cinerea*, each strain was first precultivated for 2 days at 27°C under ambient light/dark conditions. Then, both strains were inoculated onto LB agar plates by striking living cells on a line with an inoculation loop, while the controls consisted of incubating each organism alone with no other stimulus. The experiment was conducted in biological triplicates. After 8 days of incubation at 27°C, the plates were photographed, and the antibiosis areas and bands of the same size located at the edge of the axenic cultures were recovered, cut into small pieces and extracted with 100% methanol under overnight static conditions, followed by 30 min of sonication in an ultrasonic bath. The organic filtrates were passed through folded Whatman® filters (quality 595 ½, diameter 110 mm) and dried under a nitrogen flow for 2 h. The samples were then diluted in methanol:water (1:1, dilution 1/10, UHPLC-grade CHEMSOLUTE®), and 1 μL was injected into a C-18 column (Kinetex, 100 × 2.1 mm, 2.6 μm, Thermo Accucore) and analyzed with a UHPLC-HR-MS (UltiMate 3000 UHPLC, Dionex, USA) coupled to a Q-Exactive Plus Orbitrap mass spectrometer (Thermo Fisher Scientific, Dreieich, Germany). The mass spectrometry analysis was performed in both positive- and negative-ion mode with a scan range of *m*/*z* 100–1,500 for both the full-scan and the targeted data-dependent mass spectrometry experiment (ddMS, Top N). The peak resolutions were set at 70,000 and 17,500 for the full-scan and ddMS analyses, respectively. The MS/MS experiments were conducted with an isolation window of 0.4 *m*/*z* at a peak resolution of 35,000 (average NCE 15, 30, 45). The maximum ion time was set to 200 ms, and the AGC target was set to 3e6. The analytical standard was prepared in methanol:water (1:1), and a 10 μL aliquot was injected under similar conditions to those mentioned above. The raw MS data and MS/MS spectra were uploaded to the open data repository Dryad (https://www.datadryad.org/) under the https://doi.org/10.5061/dryad.0rxwdbrv4.

For GC-MS analysis, 250 mL Erlenmeyer flasks filled with 100 mL sterile LB medium were inoculated with 50 μL of a ZJU1 culture, then covered with aluminum foil and incubated at 30°C for 48 h. Volatile organic compounds were collected via solid-phase micro extraction (SPME) using 6 biological replicates, and every replicate was sampled for 1 h at room temperature. The SPME fibers and the holders were obtained from Supelco (divinylbenzene-carboxen-polydimethylsiloxane fiber, 50/30 μm DVB/CAR/PDMS, Bellefonte, PA, USA). The fibers were first conditioned according to the manufacturer's instructions. The needles were inserted into the volatile collection equipment through a hole in the aluminum foil cover. After 1 h of exposure, the fiber was introduced into the GC injector for thermal desorption and analysis of the volatile organic compounds. An ISQ LT and Trace 1310 (Thermo Fisher Scientific, Dreieich, Germany) device equipped with a ZB5 column (30 m, 0.25 mm I.D., 0.25 μm film thickness) was run connected to a guard column (10 m, Phenomenex, Aschaffenburg, Germany) using helium (1.2 mL min^−1^) as the carrier gas. Mass spectra were measured in electron impact (EI) mode at 70 eV, 41–450 *m/z*. Volatiles were eluted under the following programmed conditions: 40°C (2 min isotherm), followed by heating at 10°C min^−1^ to 200°C and at 40°C min^−1^ to 280°C (1 min isotherm). The GC injector (split ratio 1:10), transfer line and ion source were set at 230, 280, and 250°C, respectively. Compounds were identified using standards.

### Data Processing and Metabolomics Analysis

The raw data were analyzed in Compound Discoverer software 2.1 (Thermo Fisher Scientific, Dreieich, Germany) for peak picking, deconvolution and identification of the metabolites. The mass tolerance for fragment matching and composition prediction was set to 5 ppm, while the intensity tolerance and threshold were 30 and 0.1%, respectively. Peak detection was performed with a 30% intensity tolerance, signal-to-noise threshold of 3 and mass tolerance of 5 ppm. The signal-to-noise ratio was 3 for fragment matching and composition predictions. Peaks were filtered using a signal-to-noise ratio of 1.5. Raw MS/MS profiles were compared with open data repositories (Metabolika, KEGG, mzCloud) within Compound Discoverer without changing the suggested parameters of the program. Mass tolerance was set to 5 ppm for all tools used for spectrum similarity searched. The identification of surfactin was confirmed on the basis of the MS/MS fragments and retention times of the purchased standard (Sigma-Aldrich, Darmstadt, Germany). The identification of bacillibactin, bacillaene and bacilysin was accomplished by accurate mass interpretation and database matching based on previous literature (Phister et al., 2004; Hertlein et al., 2014).

## Results

### Isolation and Identification of the Endophytic Bacterium *B. amyloliquefaciens* From Mulberry Leaves

**Figure 1:**
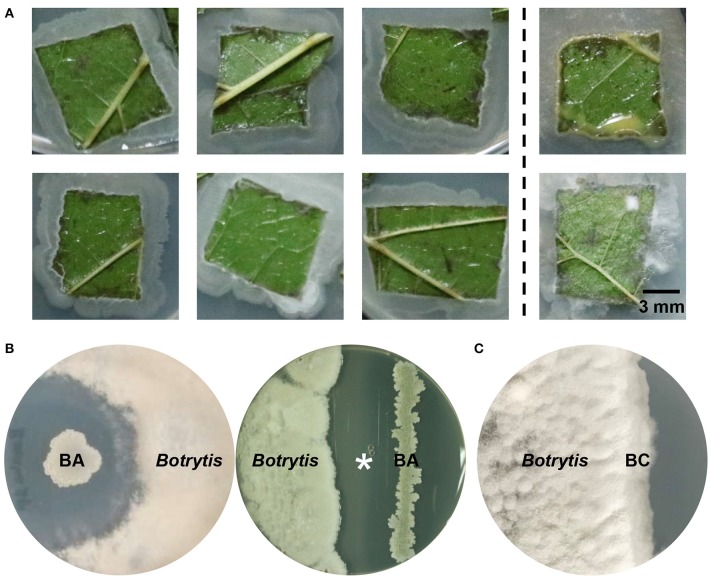
Isolation and identification of the mulberry leaf endophytic bacterium *B. amyloliquefaciens*. **(A)** Survey of indigenous bacterial endophytes from healthy mulberry leaves. Left panel, endophytes identified as *Bacillus amyloliquefaciens*; right panel, other bacterial and fungal endophytes. **(B)** Antagonistic effect of a newly isolated strain, *B. amyloliquefaciens* ZJU1 (BA), on the growth of the pathogenic fungus *Botrytis cinerea*. **(C)** The negative control *Bacillus cereus* (BC) did not show any antagonistic activity against *B. cinerea*. The asterisk indicates the competition zone extracted for the chemical detection and identification of antimicrobials.

*B. cinerea* is the most frequent fungal pathogen isolated from the mulberry-planting fields (Table S2), and causes severe anthracnose infections of mulberry. The symptoms of this mulberry gray mold disease consist of brown to black necrotic spots on the leaves of the tree, leading to yield loss of leaves for silkworm feeding and other uses. The infected leaves that fall in the field are a source of primary inoculum in the following year. To develop potential biocontrol strategies against gray mold, we screened endophytes in a mulberry field where gray mold has never been reported. Isolates from mulberry foliage were tested for their capacity to suppress *B. cinerea*. Based on colony and cell morphology, endospore formation and Gram staining, a Gram-positive, rod-shaped, endospore-forming bacterium was frequently isolated from healthy mulberry leaves (Figure 1A) and showed high efficacy in controlling gray mold caused by *B. cinerea* on agar plates under laboratory conditions (Figures 1B,C).

**Figure 2:**
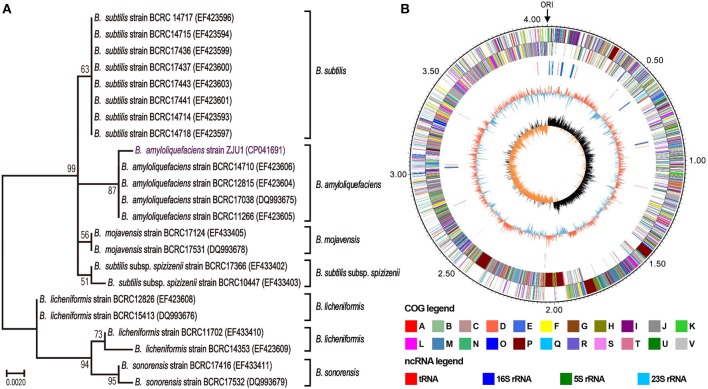
Phylogenetic analysis and circular chromosome of strain ZJU1. **(A)** Phylogenetic tree of *B. amyloliquefaciens* ZJU1 based on the 16S rRNA gene sequences of 23 representative *Bacillus* strains. BCRC, the Bioresource Collection and Research Center, Japan. The tree was built using the construct/test neighbor-joining method with bootstrap test values (based on 500 replications) expressed as a percentage of 100 at the branch points. Only bootstrap percentages above 50% are shown. **(B)** The circular genome map revealing the genetic basis of ZJU1. From outside to inside, the map shows the (1) size of the complete genome (M); (2–3) sequences encoding the amino acids in proteins on the + and – strands, with different colors representing different COG functional classifications; (4) rRNA and tRNA; (5) GC content, where the outward red portion indicates that the GC content in this region is higher than the average GC content of the whole genome, and the inward blue portion indicates that the GC content in this region is lower than the whole genome average, where the higher the peak value, the greater the difference from the average GC content; and (6) GC skew value (G–C/G+C). When the value is positive, the CDS is more likely to be transcribed from the positive chain; otherwise, the CDS is more likely to be transcribed from the negative chain.

Strain ZJU1 is a cultured representative that was initially identified by 16S rRNA analysis as *Bacillus amyloliquefaciens*. It is well-established that the species *B. amyloliquefaciens* is associated with many plants (Belbahri et al., 2017), whereas to the best of our knowledge, this is the first report of the presence of *B. amyloliquefaciens* inside woody tree leaves. Phylogenetic reconstruction based on 16S rRNA sequences also showed that ZJU1 forms a phylogenetic lineage distinct from other *B. amyloliquefaciens* strains (Figure 2A). Thus, *B. amyloliquefaciens* ZJU1 represents a novel leaf endophyte that is closely associated with healthy mulberry trees.

### Whole-Genome Sequencing of *B. amyloliquefaciens* ZJU1 and Comparative Genomic Analyses

We sequenced the ZJU1 genome to investigate the general characteristics of this *Bacillus* strain. A total of 1.15 billion high-quality bases (284-fold genome coverage) were generated via the whole-genome shotgun sequencing approach. *De novo* assembly of the sequences successfully generated a 4,064,151 bp circular chromosome with 4,144 genes, and an average gene length of 876 bp (Figure 2B), and no plasmids were detected. The total length of the genes was estimated to be 3,630,891 bp, with a 47.22% GC content, accounting for 89.34% of the entire genome (Table S3). Genome component analyses, including the prediction of genomic islands, CRISPR sequences, tRNA and rRNA, prophages, and repetitive sequences, are also shown in Figure 2B, Figure S1 and Tables S4–S7. Among these components, 9 genomic islands, 86 tRNA genes, and 27 rRNA operons were predicted in the ZJU1 chromosome.

The functional annotation of the resulting genomic sequences was based on a whole-genome BLAST search against several commonly used databases. In the COG analysis, 2,947 COGs were classified into 24 functional categories, and amino acid transport and metabolism constituted the most enriched metabolic category, including 283 related genes. The second highest percentage of COGs was in the transcription category, with 251 genes involved (Figure S2). Gene ontology (GO) analysis indicated that the genes related to metabolic process, catalytic activity and cell parts accounted for the highest proportions among the biological process, molecular function and cellular component categories, respectively (Figure S3). In the KEGG pathways, the number of genes involved in the metabolic pathways (583 unigenes), the biosynthesis of secondary metabolites (277 unigenes) and microbial metabolism in diverse environments (158 unigenes) accounted for 30.68% of the predicted genes (Table S8). Whole-genome-based phylogenetic analysis of ZJU1 revealed that it may be more appropriately identified as a *B. amyloliquefaciens* subsp. *plantarum* strain, and the most closely related strain to ZJU1 with an available genome sequence is within *B. amyloliquefaciens* Y2, which was isolated from wheat (Figure S4).

**Figure 3:**
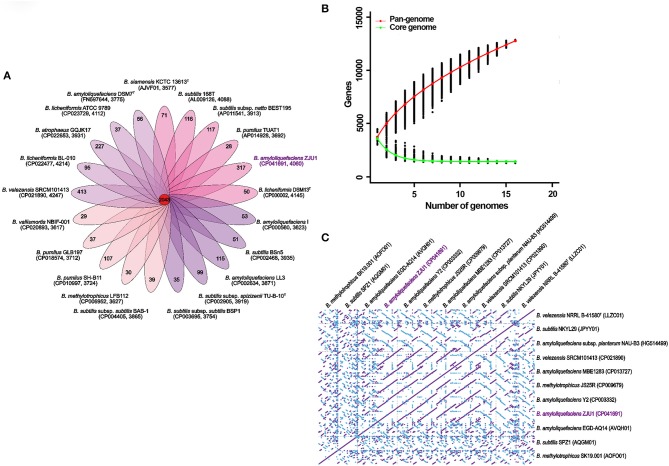
Multigenomic analysis revealing the pangenome of *Bacillus* and genome synteny. **(A)** Flower plot illustrating the number of shared (core) and specific (accessory/dispensable) genes based on clusters of orthologs. Each petal displays the number of strain-specific genes found in each genome, with the core orthologous gene number in the center. Inside the brackets, the accession number of the strain and its total number of gene families are shown. **(B)** Pan and core genome gene numbers in representative *Bacillus* taxa. The red continuous curve represents the total number of genes (pangenome) for a given number of sequentially added genomes; green indicates the number of ubiquitous genes (core genome) as a function of the number of sequentially added genomes. The vertical bars correspond to standard deviations after repeating random combinations of the genomes. **(C)** Synteny plot of *Bacillus* chromosomes based on whole-genome alignments. The diagonal lines calculated with MUMmer display the homologous regions in pairwise genome comparisons. Forward and complimentary strands between the genomic sequences are represented in purple and blue, respectively.

For comparative genomic analysis, the global gene repertoire of the genus *Bacillus* was first determined by profiling 42 representative genome assemblies (Table S9). In total, 82,950 genes were clustered from these different species into 8,804 orthologous groups (OGs) by OrthoMCL. Among these clusters, 2,093 contained only a single gene copy (singletons); 667 contained two gene copies (doublets); and 6,044 contained three or more gene copies. We found that these *Bacillus* genomes shared 2,043 gene family clusters, and strain ZJU exhibited the second largest number (317) of unique clusters after *Bacillus velezensis* SRCM101413 (413 clusters) (Figure 3A). The gene numbers corresponding to the core genome of genus *Bacillus* decreased with the addition of new strains, and the accumulation curve for the number of genes in common tended to reach a plateau with the inclusion of 16 genomes (in green, Figure 3B). In contrast to the core genome estimates, the total number of the possible genes—the pangenome—of *Bacillus* species appeared not to have been saturated, and the gene accumulation curve continued to rise, as depicted in Figure 3B (in red). Based on these genome sequences, the *Bacillus* core genome consisted of 1,263 genes, representing approximately 28.34% of the average genome size (*N* = 21) in the same genus. The pangenome for all lineages consisted of 12,889 genes, corresponding to about 3-fold the average size of these selected genomes.

To better understand the correlated gene arrangements among taxa, we examined synteny and collinearity in the *Bacillus* genomes by aligning the gene loci. Chromosomal collinearity assessed through whole-genome alignments revealed a striking level of conservation in the same direction between the genome sequence of ZJU1 and that of strain *B. amyloliquefaciens* Y2. As expected for two strains with very close phylogenetic relationships, nearly all regions of sequence similarity fell along the diagonals of the forward strand, except for the only two gene sites, indicating a generally similar gene and sequence order (Figure 3C). Similarly, the longest stretches of conserved syntenic blocks were observed between the *Bacillus amyloliquefaciens* NAU-B3 and ZJU1 genomes along the diagonals of the complimentary strand. Therefore, *B. amyloliquefaciens* NAU-B3 is most similar to ZJU1 on the reverse strand. Notably, the two strains were the same as ZJU1 belonging to the *B. amyloliquefaciens* species, but both were isolated from grass plants. Some other environmental and food product strains also showed some similarities to the ZJU1 genome, such as JPYY01 (soil), LLZC01 (river), and CP021890 (fermentation food).

### The Bacterial Endophyte ZJU1 Limits Fungal Pathogen Damage in Mulberry Tree

Genome analysis further revealed major traits of strain ZJU1 in the antagonistic effects on the growth of *B. cinerea*. There were 31 gene clusters related to secondary metabolite biosynthesis in this strain (Table S10). Among these clusters, 10 candidates covering over 689 kb in total were identified as antimicrobials, including four polyketides, three non-ribosomal peptides (NRPS), one ribosomally synthesized and posttranslationally modified peptide (RiPP), and two others (Table 1). In particular, using high-resolution mass spectrometry, we were able to characterize whether these molecules were truly secreted by ZJU1. The chemical identification of active fractions in the competition zone indicated that surfactin, bacillaene, bacillibactin, and bacilysin were specifically produced by *B. amyloliquefaciens* ZJU1 to inhibit the growth of the phytopathogenic fungus *B. cinerea* (Table 1). Moreover, gas chromatography-mass spectrometry (GC-MS) analysis showed that strain ZJU1 also emitted a cocktail of diffusible and volatile organic compounds (VOCs) with antifungal activity, including 2-heptanone, 2-nonanone, and 2-undecanone (Figure S5). Therefore, the bacterial endophyte ZJU1 presents an inherent ability to produce diverse secondary metabolites directed against *B. cinerea* and other phytopathogens.

**Table 1:** Gene clusters directing antimicrobial synthesis in *B. amyloliquefaciens* ZJU1 and the *in situ* determination of metabolites.

**Type**	**From**	**To**	**Most similar known cluster**	**Similarity (%)**	**Molecular formula**	***m/z* [M+H]^**+**^**	**Search MS/MS (min)**
NRPS	314,623	379,675	**Surfactin**	82	C_53_H_93_N_7_O_13_	1036.6904	4.27
Other	718,721	748,313	Plantathiazolicin/ plantazolicin				
PKS-like	942,322	983,566	Butirosin	7	–	–	–
TransAT-PKS	1,439,711	1,527,545	Macrolactin	100	–	–	–
TransAT-PKS, transAT-PKS-like, NRPS	1,749,663	1,858,957	**Bacillaene**	100	C_34_H_48_N_2_O_6_	581.3585	2.99
NRPS, transAT-PKS, betalactone	1,926,173	2,060,851	Fengycin	100			
TransAT-PKS-like, transAT-PKS	2,361,210	2,467,383	Difficidin	100	–	–	–
NRPS, bacteriocin	3,105,153	3,155,662	**Bacillibactin**	100	C_39_H_42_N_6_O_18_	883.2628	2.38
Other	3,707,421	3,748,839	**Bacilysin**	100	C_12_H_18_N_2_O_5_	271.1288	0.73 (1.8, another m/z 271.1758)
Lantipeptide	3,903,898	3,927,086	Mersacidin				

**Figure 4:**
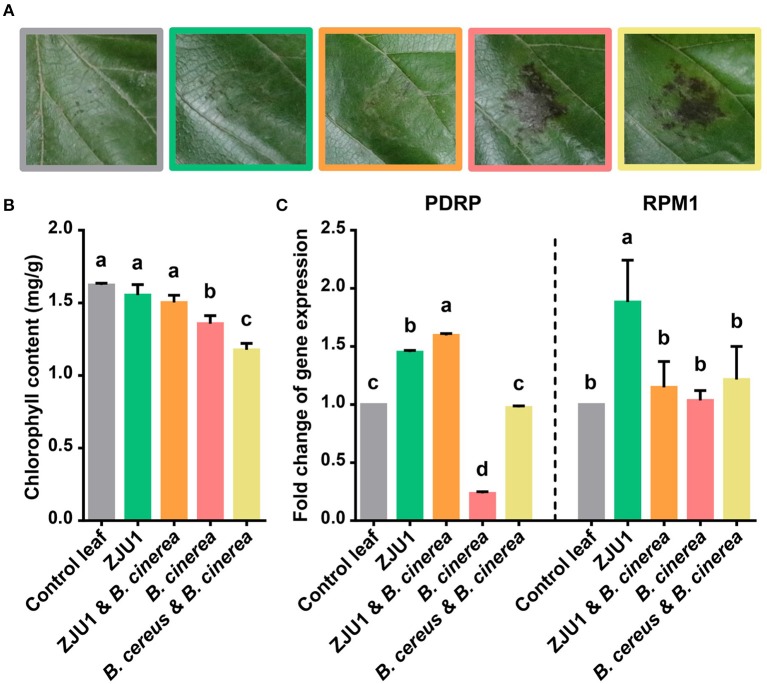
The bacterial endophyte ZJU1 limits fungal pathogen damage in mulberry. **(A)** Anthracnose disease inhibition by strain ZJU1 on mulberry leaves. Representative results of at least five independent experiments are shown. Same color key as in **(B,C)**. **(B)** Leaf chlorophyll content indicating the physiological status of plants under different treatments. **(C)** Expression of putative disease resistance protein-encoding genes of *Morus*. PDRP, putative disease resistance protein SUMM2; RPM1, disease resistance protein RPM1. Different letters indicate a significant (*P* < 0.05) difference between treatments by one-way analysis of variance. Error bars indicate ±SD of five replicates.

To validate its use in controlling plant disease under field conditions, ZJU1 was tested *in planta* on mulberry leaves together with the negative control bacterium *Bacillus cereus* (Figure 4A). The negative control *B. cereus*, a common commensal bacillus species isolated from the same field, appeared not to increase plant performance or affect the pathogenicity of *B. cinerea*; the infection of plants with *B. cinerea* caused typical disease symptoms. By contrast, the endophyte ZJU1 strongly inhibited the mycelial growth and conidial germination of the fungal pathogen and reduced the incidence or severity of leaf disease in mulberry. Since leaf chlorophyll content provides valuable information about the physiological status of plants, we investigated the chlorophyll content of mulberry leaves after pathogen challenge. The measurement of chlorophyll content showed that chlorophyll was lost more quickly in *B. cinerea*-infected and *B. cereus* coinoculated plants; however, ZJU1-coinoculated plants suffered less, thereby maintaining their photosynthetic capacity and preventing pathogen-induced leaf senescence (Figure 4B). These results demonstrated that the bacterial endophyte ZJU1 is sufficient to limit fungal pathogen damage in mulberry plants.

In addition to the direct antagonism of pathogens, other mechanisms of protection may also exist, such as the stimulation of systemic host responses. We further characterized the mulberry disease resistance gene expression profiles from the *in planta* experiments. Consistent with previous reports, the plant pathogen *B. cinerea* reduced the expression level of the putative *Morus* disease resistance protein (PDRP) after infection. In contrast, the transcript abundance of pathogen defense marker genes (PDRP and RPM1) increased significantly in the presence of ZJU1 (Figure 4C). *B. cereus* did not significantly enhance the expression level of resistance genes.

Taken together, these results suggest that the production of diverse antagonistic metabolites and activation of the plant defense pathway jointly contribute to the control of the fungal pathogen in mulberry trees.

### Strain ZJU1 Antagonizes the Herbivorous Insect Pest *S. litura*

**Figure 5:**
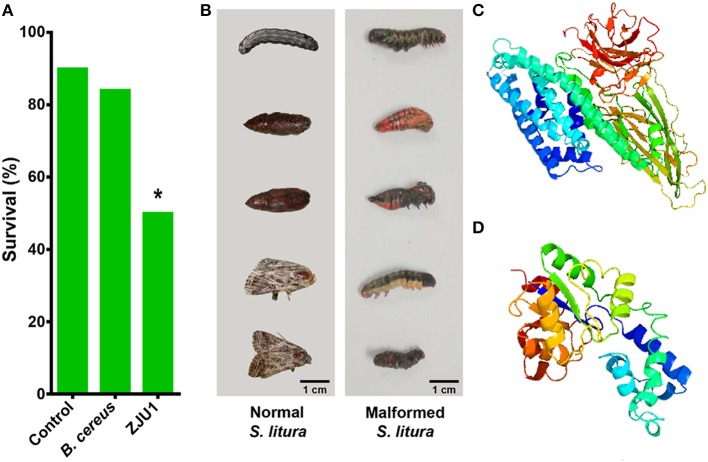
Strain ZJU1 antagonizes the herbivorous insect pest *S. litura*. **(A)** Oral insecticidal activity of strain ZJU1. **(B)** Left panel, normal *S. litura* not exposed to strain ZJU1; right panel, larval and pupal deformities caused by ZJU1. **(C)** Tertiary structure prediction for Cry10Aa of *B. thuringiensis* and **(D)** *B. amyloliquefaciens* by Phyre2. Image colored by the rainbow N/C terminus. *Represents a significant difference.

In addition to the diverse gene clusters involved in the production of antimicrobial metabolites, the *in silico* analysis of genomic sequences revealed that *B. amyloliquefaciens* ZJU1 also harbors genes related to the biosynthesis of toxins, particularly the crystal (Cry) proteins that target herbivorous insects. Various *Bacillus* spp. have evolved specific Cry toxins to colonize insects, which are now being been widely used as a biocontrol strategy in transgenic and organic farming (Castillo-Esparza et al., 2019). Since the main insect pests of mulberry are lepidopteran herbivores, a biocontrol trial with the ZJU1 strain has been performed in the devastating agricultural pest *S. litura* (Lepidoptera: Noctuidae). The insecticidal activity of ZJU1 was proven via the oral infection of *S. litura* larvae. The feeding assay showed that the average survival rate of the *Spodoptera* larvae was below 50% after exposure to *B. amyloliquefaciens* ZJU1 (Figure 5A). Meanwhile, the untreated control larvae and *B. cereus*-treated group exhibited higher survival rates (90 and 84%, respectively) during development into pupae. ZJU1 resulted in disease symptoms, pupal malformation, and high mortality (Figure 5B), as described in other lepidopterans infected by Gram-positive entomopathogens (Shao et al., 2017).

We compared the sequence of the Cry toxin gene in *B. amyloliquefaciens* ZJU1 with all sequences in the NCBI database, which revealed that this gene is most similar to the Cry10Aa gene from *B. thuringiensis*. A further comparison of the stereo structures of the Cry10Aa protein from the two *Bacillus* species provided insight into some important characteristics of this kind of pesticidal crystal toxin. We found that both Cry10Aa proteins are mostly composed of α-helix and β-sheet structures (Figures 5C,D). Although the protein of *B. thuringiensis* (Figure 5C) has a more complex structure than that in *B. amyloliquefaciens* ZJU1 (Figure 5D), interestingly, a higher proportion of α-helix (53%) structures was found in the Cry10Aa of *B. amyloliquefaciens* ZJU1 compared to that of *B. thuringiensis* (only 32%). Considering that Cry toxins have been successfully used as a bioinsecticide against caterpillars, beetles, mosquitoes, and flies, the new Cry toxin identified from the mulberry endophyte ZJU1 not only offers another positive effect on host plants but also represents a novel biocontrol agent in biotechnology.

## Discussion

The development and application of microbial agents for the biocontrol of diseases and pests has received a significant amount of interest in recent years (Xie et al., 2019). In particular, native isolates offer environmentally friendly alternatives to chemical germicides and pesticides and are commonly accepted compared to genetically modified biocontrols. Endophytes living inside plants exhibit an intimate and often symbiotic interaction with their hosts, thus representing a valuable resource for the screening microbial agents with biotechnological potential. To date, only a few plants have ever been completely studied relative to their endophytic biology; consequently, the opportunity to find new and beneficial bacterial endophytes is considerable among the diverse plants in different ecosystems. In this study, we systematically investigated the leaf endophytes of mulberry, an ecologically, economically, and medicinally important plant, and identified a novel *Bacillus* strain ZJU1 that is a promising candidate for the development of biocontrol agents and biotechnology innovation.

The genus *Bacillus* comprises a physiologically versatile group of bacteria that includes strains isolated from diverse habitats such as food products, soil, rhizosphere and plant tissue (Vallet et al., 2017). *Bacillus* has been recognized as a good option for biocontrol applications. Most of their registered products (e.g., in the EU pesticide database) are based on *Bacillus* (Berg et al., 2017). There are a number of different reasons for their prevalent use: they present advantages over Gram-negative bacteria due to spores they form, allowing them to survive unfavorable conditions; they are easy to formulate and exhibit a prolonged shelf-life and high temperature stability (Wu et al., 2018; Xu et al., 2018; Zhang et al., 2018). *Bacillus* species such as *Bacillus licheniformis, Bacillus cereus, Bacillus subtilis, Bacillus coagulans*, and *Bacillus clausii* are already used as bacterial antagonists of plant pathogens and as plant growth-promoting bacteria (Cisternas-Jamet et al., 2019; Gautam et al., 2019). To better understand the relationship between *B. amyloliquefaciens* ZJU1 and other *Bacillus* strains, we performed comparative genomic analysis of the 21 representative strains in this work, which revealed a high level of conservation among the genomes. The genes of the core genome present in all strains are essential to the *Bacillus* life cycle, traits for habitat adaptation and biotechnological potential. Despite the limited number of strains employed here, this result is also consistent with previous studies on a broad range of *Bacillus* taxa (Alcaraz et al., 2010; Kim et al., 2017). Notably, ZJU1, showing the highest degree of collinearity with *Bacillus amyloliquefaciens* Y2, exhibits relatively greater differences in gene contents (317 non-shared family clusters) that may herald its unusual biological potential. Rarefaction analysis of pangenomes further reflected a tremendous increase in strain-specific new traits, indicating the wide diversity of biological functions harbored by *Bacillus* species. Clearly, more sequencing efforts of new strains are necessary to better understand the genomic structures and diversity of these agriculturally and industrially important bacteria.

Genome sequencing and analysis of *B. amyloliquefaciens* ZJU1 revealed that it encodes an impressive arsenal of antimicrobial compounds, among which four products exhibiting highly antifungal activity were detected *in situ* (Loeffler et al., 1986; Chen et al., 2009; Um et al., 2013; Li et al., 2014; Chen K. et al., 2018). In particular, non-ribosomally synthesized lipopeptides and polyketides have been demonstrated to make a significant contribution to plant disease protection (Molinatto et al., 2017). In addition, ZJU1 can produce a variety of bioactive VOCs to antagonize phytopathogens. Among these compounds, 2-heptanone and 2-nonanone were shown to exhibit strong antifungal properties (Wu et al., 2019), suggesting that VOCs produced by ZJU1 also play a role in the process of biocontrol. Our *in planta* experiments finally verified that the occurrence of gray mold disease caused by the major mulberry pathogen *B. cinerea* could be successfully suppressed through the application of ZJU1. In addition to controlling diseases directly through its antimicrobial activity, ZJU1 stimulates the expression of immune-related genes in plants and prevents its hosts from continuing to deteriorate under the action of *B. cinerea*. At the same time, *B. cinerea* significantly suppressed the host immune response, a well-known virulence strategy for pathogen invasion (Xin et al., 2018). In the interaction between *Arabidopsis* leaves and the plant-beneficial *Bacillus cereus* strain AR156, genes involved in several defense pathways, such as salicylate- and jasmonate/ethylene-dependent signaling pathways, were upregulated (Lopes et al., 2018), indicating that beneficial microorganisms can manipulate host immunity to establish a successful relationship with the host. Altogether, these results demonstrate that ZJU1 protects plants against pathogens not only by directly reducing pathogen growth but also by indirectly inducing plant systemic resistance.

A variety of *Bacillus* species, particularly *B. thuringiensis*, have been reported to exhibit insecticidal potential involving several mechanisms, including the production of crystal toxins such as the Cry and Cyt proteins (Berry and Crickmore, 2017). However, to the best of our knowledge, no studies have reported any *B. amyloliquefaciens* strain Cry toxin genes to date. Here, a Cry gene was detected in the strain ZJU1 for the first time. This Cry10Aa gene encodes a completely different insecticidal protein compared to those encoded by other entomopathogenic *Bacillus* spp., and notably, the Cry10Aa toxin of ZJU1 has a compact structure and, hence, a more stable configuration (53% alpha helix ratio) than its close relative in *B. thuringiensis* (Pardo-López et al., 2013). Furthermore, our feeding experiment validated that *B. amyloliquefaciens* ZJU1 had negative effects on the generalist lepidopteran pest *S. litura* growing from larva to pupa, thereby positively affecting plant development. These results, taken together, highlight that the antagonistic bacterium ZJU1 can also serve as a new microbial insecticide against widespread lepidopteran pests and can broaden the application of crystal toxins in genetically modified crops to support sustainable agriculture.

In conclusion, the leaf endophyte *B. amyloliquefaciens* ZJU1 isolated from mulberry tree not only represents a novel plant-beneficial bacterium but also possesses great potential for use in bioengineering and biotechnology. Our systemic genomic and functional characterization provides valuable and comprehensive information and will facilitate its wider effective application. Clearly, the production of all of these antibiotic and insecticidal compounds suggests that *B. amyloliquefaciens* ZJU1 is a good candidate for the development of biocontrol strategies against emerging gray mold-related infectious diseases and notorious lepidopteran pests, for instance, through artificial inoculation of the soil/rhizosphere or seeds/seedlings and direct injection into plant tissues. Considering the complete genome sequence reported here and its amenability to genetic manipulation, ZJU1 could be further engineered to increase plant performance, as it was recently shown that engineering of the banana endosphere microbiome improved *Fusarium* wilt resistance in banana (Anderson et al., 2019; Liu et al., 2019). Our study therefore highlights that expanding the investigation of the plant microbiome could lead to additional successful discoveries of unexplored novel microbial symbionts with biotechnological potential.

## Data Availability Statement

The raw dataset, including both single and paired end reads (average read length of 7,482.9 bp) is deposited at DDBJ/EMBL/GenBank under BioProject ID: PRJNA544619, SRR9125050.

## Author Contributions

YS and XL designed the experiments. SX, XZ, and BC performed the genomic and biological experiments. MV, MK, AD, and WB performed the chemical work and analyzed the data. CS assisted with the bacterial isolation and chlorophyll content measurement. SX, MV, MK, and YS wrote the manuscript with contributions from all authors.

### Conflict of Interest

The authors declare that the research was conducted in the absence of any commercial or financial relationships that could be construed as a potential conflict of interest.
