# Supplementary material for: Biocontrol Potential of a Novel Endophytic Bacterium From Mulberry (Morus) Tree
Source: Front Bioeng Biotechnol. 2020 Jan 23;7:488. doi: 10.3389/fbioe.2019.00488 (PMC6990687; doi:10.3389/fbioe.2019.00488)
Supplement: Supplementary file 1 [file Data_Sheet_1.docx]

**Figure S1** Gene island prediction. Software IslandPATH in blue; SIGI-HMM in yellow; IslandPick in green; red color represents the integration result of different software predictions. The peak plot shows the distribution of GC content.


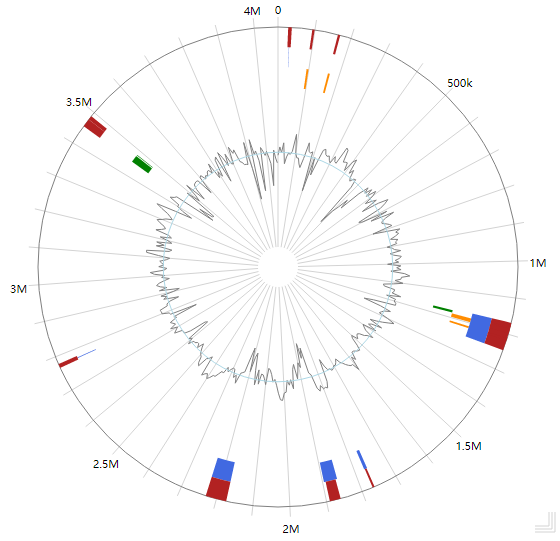


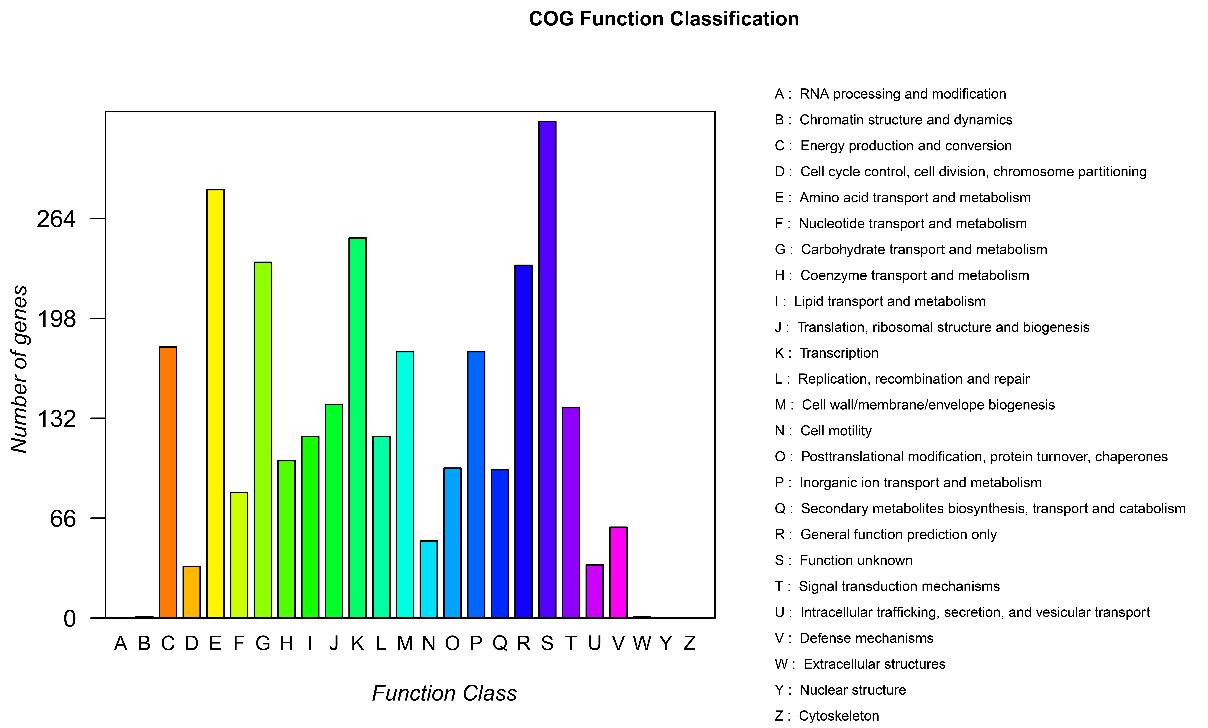
**Figure S2** COG function classifications.


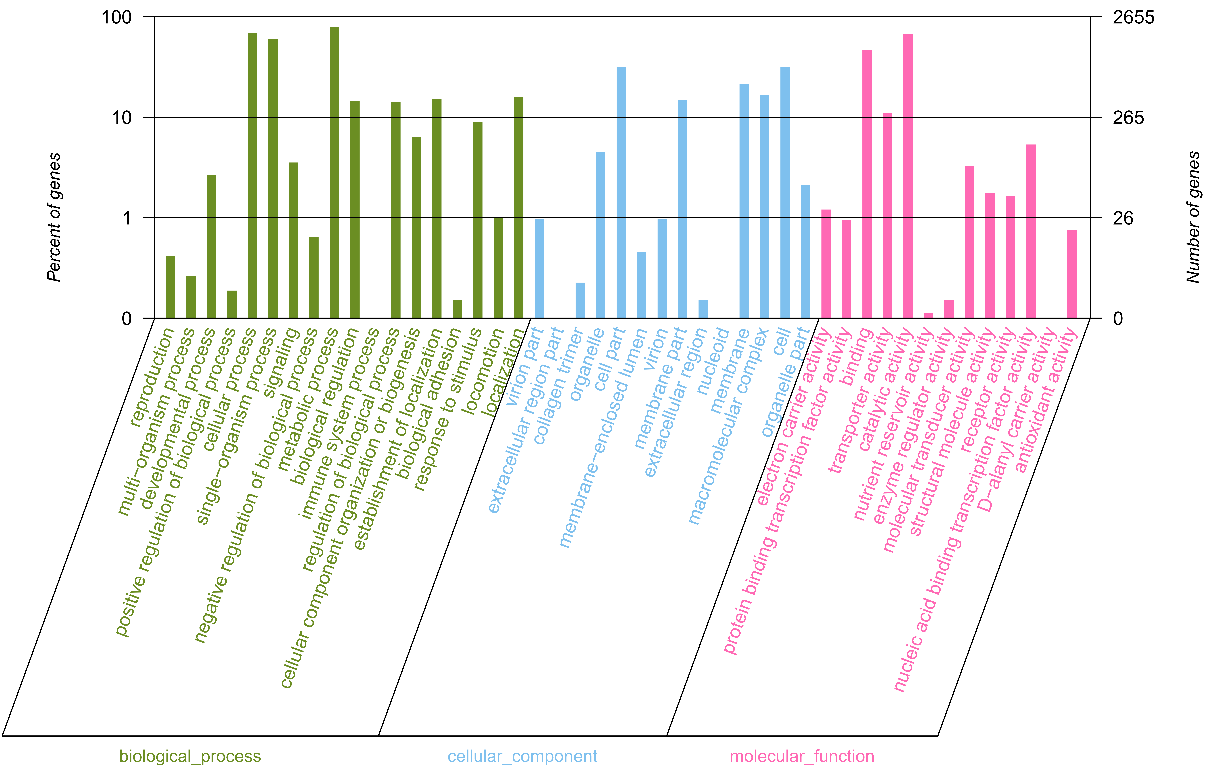
**Figure S3** Gene Ontology (GO) analysis.


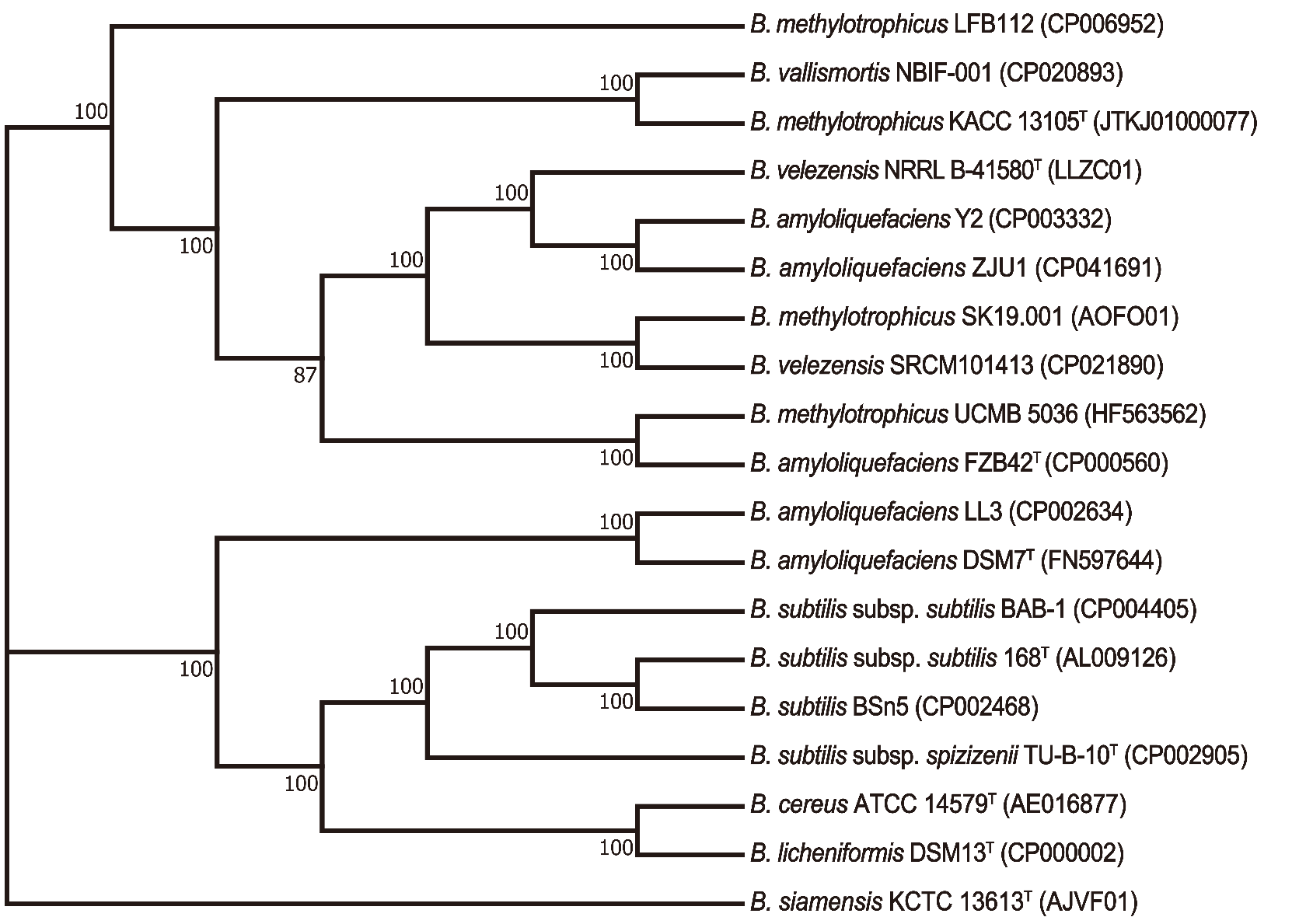
**Figure S4** Phylogenetic analysis of *B. amyloliquefaciens* ZJU1 based on the whole genomes of 19 representative *Bacillus* strains. The percentage of replicate trees in which the associated taxa clustered together in the bootstrap test (500 replicates) is shown next to the branches.

**Figure S5** (A) Schematic diagrams of volatile organic compounds (VOCs) detection. (B) Main VOCs produced by ZJU1 during incubation at 30°C for 2 d. The proportion of VOCs is shown in the pie chart. (C) Chromatogram of GC-MS measurement of main VOCs.

**Table S1** The primer sequences for amplified genes in Quantitative RT-PCR.

| ***Morus* putative disease resistance protein PDRP** | Morus010201 F | GTATTCAATTCTTGGCACT | https://www.ncbi.nlm.nih.gov/nucleotide/703072809?report=genbank&log$=nucltop&blast_rank=1&RID=0647HDGH015 | XM_010091081 |
| --- | --- | --- | --- | --- |
|  | Morus010201 R | TAAATCGATCAATGCGTCAC |  |  |
|  |  |  |  |  |
| ***Morus* putative disease resistance protein RPM1** | Morus019335 F | GTAGGCAATTTGTTCCAC | https://www.ncbi.nlm.nih.gov/nucleotide/703081650?report=genbank&log$=nucltop&blast_rank=1&RID=063HAEH1014 | XM_010093445 |
|  | Morus019335 R | ATTCATCTTTACACCACGAA |  |  |

**Table S2** Fungi isolated from mulberry leaves.

| **Total No.** | **Closest isolate match** | **%ID** | **Plant pathogen** |
| --- | --- | --- | --- |
| 1 | *Botrytis cinerea* strain SCB2-4 | 99% | Y |
| 2 | *Botrytis cinerea* isolate RP434_2 | 99% | Y |
| 3 | *Botrytis cinerea* strain SCB2-4 | 99% | Y |
| 4 | *Botryotinia fuckeliana* 18S rRNA gene | 99% | Y |
| 5 | *Botrytis cinerea* strain SMGM003 | 99% | Y |
| 6 | *Botryotinia fuckelian*a strain BGM-50 | 99% | Y |
| 7 | *Botrytis cinerea* isolate HLS-002 | 99% | Y |
| 8 | *Botrytis cinerea* strain SMGM003 | 99% | Y |
| 9 | *Botrytis cinerea* isolate RP434_2 | 100% | Y |
| 10 | *Cladosporium* sp. isolate MC-20-R | 99% | Y |
| 12 | *Cladosporium* *cladosporioides* isolate ZJDF | 100% | Y |
| 13 | *Cladosporium cladosporioides* voucher BJ1-10 | 99% | Y |
| 14 | *Dothideomycetes* sp. | 99% | Y |
| 15 | Uncultured *Cladosporium* clone | 99% | Y |
| 16 | *Dothideomycetes* sp. MSEF4 | 99% | Y |
| 17 | Uncultured *Cladosporium* clone wzl015 | 99% | Y |
| 18 | Uncultured fungus clone LX040429-122-035-H02 | 99% | Unknown |
| 19 | *Pleosporales* sp. clone CD-31 | 99% | Unknown |
| 20 | Fungal sp. strain Xmf154 | 99% | Unknown |
| 21 | Fungal sp. strain Xmf154 | 99% | Unknown |

**Table S3** Genomic features of strain ZJU1.

| **Sample** | **genome** |
| --- | --- |
| No. of all scaffolds | 1 |
| Bases in all scaffolds(bp) | 4064151 |
| No. of large scaffords(> 1000 bp) | 1 |
| Bases in large scaffolds(bp) | 4064151 |
| Largest length(bp) | 4064151 |
| Scaffold N50(bp) | 4064151 |
| Scaffold N90(bp) | 4064151 |
| G+C content(%) | 46.449 |
| N rate(%) | 0 |
| Gene num | 4144 |
| Gene total length(bp) | 3630891 |
| Gene average length(bp) | 876.18 |
| Gene density(kb) | 1.02 |
| GC content in gene region(%) | 47.22 |
| Gene/Genome(%) | 89.34 |
| Intergenetic region length(bp) | 433260 |
| GC content in intergenetic region(%) | 40.01 |
| Intergenetic length/Genome(%) | 10.66 |

**Table S4** tRNA and rRNA predicted from genome sequence of ZJU1.

| **tRNA** | **tRNA Begin** | **Bounds End** | **tRNA type** | **Anti Codon** | **Intron Bounds Begin** | **Cove End** | **Score** |
| --- | --- | --- | --- | --- | --- | --- | --- |
| 1 | 11098 | 11174 | Ile | GAT | 0 | 0 | 102.39 |
| 2 | 11189 | 11264 | Ala | TGC | 0 | 0 | 92.94 |
| 3 | 23173 | 23265 | Ser | TGA | 0 | 0 | 72.49 |
| 4 | 32835 | 32911 | Ile | GAT | 0 | 0 | 102.39 |
| 5 | 32926 | 33001 | Ala | TGC | 0 | 0 | 92.94 |
| 6 | 70904 | 70980 | Met | CAT | 0 | 0 | 83.99 |
| 7 | 70990 | 71064 | Glu | TTC | 0 | 0 | 70.77 |
| 8 | 96059 | 96134 | Val | TAC | 0 | 0 | 96.46 |
| 9 | 96140 | 96215 | Thr | TGT | 0 | 0 | 92.93 |
| 10 | 96252 | 96327 | Lys | TTT | 0 | 0 | 98.67 |
| 11 | 96334 | 96415 | Leu | TAG | 0 | 0 | 71.53 |
| 12 | 96440 | 96514 | Gly | GCC | 0 | 0 | 94.93 |
| 13 | 96529 | 96614 | Leu | TAA | 0 | 0 | 68.21 |
| 14 | 96626 | 96702 | Arg | ACG | 0 | 0 | 81.43 |
| 15 | 96718 | 96794 | Pro | TGG | 0 | 0 | 94.72 |
| 16 | 96799 | 96874 | Ala | TGC | 0 | 0 | 92.94 |
| 17 | 166556 | 166630 | Asn | GTT | 0 | 0 | 88.32 |
| 18 | 166633 | 166705 | Thr | GGT | 0 | 0 | 75.2 |
| 19 | 167047 | 167121 | Gly | GCC | 0 | 0 | 90.25 |
| 20 | 167154 | 167230 | Arg | ACG | 0 | 0 | 81.43 |
| 21 | 167247 | 167323 | Pro | TGG | 0 | 0 | 94.72 |
| 22 | 193704 | 193775 | Glu | TTC | 0 | 0 | 70.15 |
| 23 | 193779 | 193854 | Val | TAC | 0 | 0 | 96.46 |
| 24 | 193860 | 193932 | Thr | TGT | 0 | 0 | 84.64 |
| 25 | 193952 | 194036 | Tyr | GTA | 0 | 0 | 78.86 |
| 26 | 194041 | 194112 | Gln | TTG | 0 | 0 | 67.67 |
| 27 | 487708 | 487782 | Asn | GTT | 0 | 0 | 87.08 |
| 28 | 487786 | 487876 | Ser | GCT | 0 | 0 | 71.43 |
| 29 | 487896 | 487970 | Glu | TTC | 0 | 0 | 78.45 |
| 30 | 487982 | 488056 | Gln | TTG | 0 | 0 | 75.97 |
| 31 | 488090 | 488165 | Lys | TTT | 0 | 0 | 98.67 |
| 32 | 488174 | 488255 | Leu | TAG | 0 | 0 | 73.07 |
| 33 | 488324 | 488407 | Leu | GAG | 0 | 0 | 53.11 |
| 34 | 596209 | 596285 | Arg | ACG | 0 | 0 | 80.45 |
| 35 | 596294 | 596367 | Gly | TCC | 0 | 0 | 78.55 |
| 36 | 601306 | 601382 | Met | CAT | 0 | 0 | 90.17 |
| 37 | 601389 | 601465 | Asp | GTC | 0 | 0 | 95.8 |
| 38 | 877227 | 877301 | Asn | GTT | 0 | 0 | 88.32 |
| 39 | 877307 | 877397 | Ser | GGA | 0 | 0 | 61.49 |
| 40 | 877434 | 877505 | Glu | TTC | 0 | 0 | 70.15 |
| 41 | 877516 | 877591 | Val | TAC | 0 | 0 | 96.46 |
| 42 | 877599 | 877675 | Met | CAT | 0 | 0 | 90.17 |
| 43 | 877686 | 877762 | Asp | GTC | 0 | 0 | 95.8 |
| 44 | 877773 | 877848 | Phe | GAA | 0 | 0 | 97.89 |
| 45 | 877852 | 877924 | Thr | TGT | 0 | 0 | 82.36 |
| 46 | 877936 | 878020 | Tyr | GTA | 0 | 0 | 78.86 |
| 47 | 878031 | 878104 | Trp | CCA | 0 | 0 | 71.01 |
| 48 | 878134 | 878209 | His | GTG | 0 | 0 | 72.51 |
| 49 | 878219 | 878290 | Gln | TTG | 0 | 0 | 67.67 |
| 50 | 878344 | 878418 | Gly | GCC | 0 | 0 | 90.25 |
| 51 | 878425 | 878495 | Cys | GCA | 0 | 0 | 67.07 |
| 52 | 878503 | 878591 | Leu | TAA | 0 | 0 | 76.51 |
| 53 | 878837 | 878918 | Leu | CAA | 0 | 0 | 61.23 |
| 54 | 898936 | 899009 | Gly | TCC | 0 | 0 | 78.55 |
| 55 | 1169363 | 1169435 | Val | GAC | 0 | 0 | 71.64 |
| 56 | 2537903 | 2537973 | Gln | TTG | 0 | 0 | 65.47 |
| 57 | 3998557 | 3998482 | Lys | TTT | 0 | 0 | 98.67 |
| 58 | 3998473 | 3998402 | Glu | TTC | 0 | 0 | 70.15 |
| 59 | 3998317 | 3998241 | Asp | GTC | 0 | 0 | 95.8 |
| 60 | 3998204 | 3998132 | Phe | GAA | 0 | 0 | 89.6 |
| 61 | 3384536 | 3384461 | Arg | CCG | 0 | 0 | 66.66 |
| 62 | 3043469 | 3043397 | Ala | GGC | 0 | 0 | 77.43 |
| 63 | 3025408 | 3025333 | Val | TAC | 0 | 0 | 96.46 |
| 64 | 3025303 | 3025228 | Thr | TGT | 0 | 0 | 91.03 |
| 65 | 3025190 | 3025115 | Lys | TTT | 0 | 0 | 98.67 |
| 66 | 3025104 | 3025021 | Leu | CAG | 0 | 0 | 64.19 |
| 67 | 3025011 | 3024937 | Gly | GCC | 0 | 0 | 90.25 |
| 68 | 3024917 | 3024832 | Leu | TAA | 0 | 0 | 68.21 |
| 69 | 3024822 | 3024746 | Arg | ACG | 0 | 0 | 81.43 |
| 70 | 3024729 | 3024653 | Pro | TGG | 0 | 0 | 94.72 |
| 71 | 3024648 | 3024576 | Ala | TGC | 0 | 0 | 84.64 |
| 72 | 3024555 | 3024479 | Met | CAT | 0 | 0 | 83.99 |
| 73 | 3024476 | 3024400 | Met | CAT | 0 | 0 | 97.54 |
| 74 | 3024392 | 3024300 | Ser | TGA | 0 | 0 | 72.49 |
| 75 | 3024280 | 3024204 | Met | CAT | 0 | 0 | 90.17 |
| 76 | 3024197 | 3024121 | Asp | GTC | 0 | 0 | 95.8 |
| 77 | 3024110 | 3024035 | Phe | GAA | 0 | 0 | 97.89 |
| 78 | 3024014 | 3023939 | His | GTG | 0 | 0 | 72.51 |
| 79 | 3023930 | 3023857 | Gly | TCC | 0 | 0 | 81.36 |
| 80 | 3023841 | 3023765 | Ile | GAT | 0 | 0 | 102.39 |
| 81 | 3023754 | 3023680 | Asn | GTT | 0 | 0 | 87.08 |
| 82 | 3023676 | 3023586 | Ser | GCT | 0 | 0 | 72.43 |
| 83 | 3023582 | 3023511 | Glu | TTC | 0 | 0 | 70.15 |
| 84 | 2778108 | 2778035 | Arg | TCT | 0 | 0 | 81.02 |
| 85 | 2777910 | 2777837 | Arg | TCT | 0 | 0 | 81.02 |
| 86 | 2049115 | 2049043 | Arg | CCT | 0 | 0 | 69.33 |

| **Sequence name** | **Begin** | **End** | **+/-** | **Attribute** |
| --- | --- | --- | --- | --- |
| ZJU1 | 9450 | 10997 | + | 16S_rRNA |
| ZJU1 | 11349 | 14274 | + | 23S_rRNA |
| ZJU1 | 14329 | 14439 | + | 5S_rRNA |

**Table S5** Repetitive sequences predicted from genome sequence of ZJU1.

| **Sequence name** | **Type** | **Number (#)** | **Total length (bp)** | **In genome (%)** |
| --- | --- | --- | --- | --- |
| ZJU1 | tandem repeat | 26 | 7325 | 0.0018 |

**Table S6** Genomic islands prediction in the ZJU1 genome sequence.

| **Island start** | **Island end** | **Length** | **Gene ID** | **Locus** | **Gene start** | **Gene end** | **Strand** | **Product** |
| --- | --- | --- | --- | --- | --- | --- | --- | --- |
| 27590 | 37312 | 9722 |  | orf0022 | 27590 | 27715 | -1 | MULTISPECIES: hypothetical protein |
| 27590 | 37312 | 9722 | dnaX | orf0023 | 27719 | 29410 | 1 | MULTISPECIES: DNA polymerase III subunit gamma/tau |
| 27590 | 37312 | 9722 |  | orf0024 | 29456 | 29758 | 1 | MULTISPECIES: hypothetical protein |
| 27590 | 37312 | 9722 | recR | orf0025 | 29773 | 30369 | 1 | MULTISPECIES: recombinase RecR recombination protein RecR |
| 27590 | 37312 | 9722 |  | orf0026 | 30388 | 30612 | 1 | MULTISPECIES: hypothetical protein |
| 27590 | 37312 | 9722 | bofA | orf0027 | 30673 | 30936 | 1 | MULTISPECIES: sigma-K factor-processing regulatory protein BofA |
| 27590 | 37312 | 9722 |  | orf0028 | 33042 | 33515 | -1 | hypothetical protein BSNT_06294 |
| 27590 | 37312 | 9722 |  | orf0029 | 36354 | 36548 | 1 | MULTISPECIES: CsfB |
| 27590 | 37312 | 9722 |  | orf0030 | 36698 | 37312 | 1 | MULTISPECIES: protein xpaC |
| 92898 | 99255 | 6357 |  | orf0089 | 92898 | 93371 | -1 | hypothetical protein BSNT_06294 |
| 92898 | 99255 | 6357 |  | orf0090 | 98782 | 99255 | -1 | hypothetical protein BSNT_06294 |
| 163372 | 169782 | 6410 |  | orf0159 | 163372 | 163845 | -1 | hypothetical protein BSNT_06294 |
| 163372 | 169782 | 6410 |  | orf0160 | 169309 | 169782 | -1 | hypothetical protein BSNT_06294 |
| 1168048 | 1245311 | 77263 | metB | orf1200 | 1166934 | 1168055 | 1 | cystathionine gamma-synthase |
| 1168048 | 1245311 | 77263 | metC | orf1201 | 1168048 | 1169223 | 1 | cystathionine beta-lyase |
| 1168048 | 1245311 | 77263 | xerD | orf1202 | 1169595 | 1170641 | 1 | site-specific integrase |
| 1168048 | 1245311 | 77263 |  | orf1203 | 1171275 | 1171724 | 1 | MULTISPECIES: hypothetical protein |
| 1168048 | 1245311 | 77263 |  | orf1204 | 1173010 | 1173513 | 1 |  |
| 1168048 | 1245311 | 77263 |  | orf1205 | 1173725 | 1174090 | 1 | hypothetical protein |
| 1168048 | 1245311 | 77263 |  | orf1206 | 1174087 | 1174455 | 1 | hypothetical protein |
| 1168048 | 1245311 | 77263 |  | orf1207 | 1174674 | 1175858 | 1 | hypothetical protein |
| 1168048 | 1245311 | 77263 |  | orf1208 | 1175855 | 1176034 | 1 | hypothetical protein |
| 1168048 | 1245311 | 77263 |  | orf1209 | 1176027 | 1176806 | 1 | DNA replication protein |
| 1168048 | 1245311 | 77263 |  | orf1210 | 1176803 | 1177312 | 1 | hypothetical protein |
| 1168048 | 1245311 | 77263 | dnaB | orf1211 | 1177309 | 1178724 | 1 | DNA helicase |
| 1168048 | 1245311 | 77263 |  | orf1212 | 1178859 | 1179854 | 1 | DNA primase |
| 1168048 | 1245311 | 77263 |  | orf1213 | 1179871 | 1180026 | -1 | Xre family transcriptional regulator |
| 1168048 | 1245311 | 77263 |  | orf1214 | 1180170 | 1180379 | -1 | hypothetical protein |
| 1168048 | 1245311 | 77263 |  | orf1215 | 1180493 | 1181719 | 1 | hypothetical protein |
| 1168048 | 1245311 | 77263 |  | orf1216 | 1181978 | 1182613 | 1 | hypothetical protein |
| 1168048 | 1245311 | 77263 |  | orf1217 | 1182758 | 1182919 | 1 | hypothetical protein A943_08050 |
| 1168048 | 1245311 | 77263 |  | orf1218 | 1182916 | 1183482 | 1 | hypothetical protein |
| 1168048 | 1245311 | 77263 |  | orf1219 | 1183720 | 1184475 | 1 | single-stranded DNA-binding protein |
| 1168048 | 1245311 | 77263 |  | orf1220 | 1184510 | 1184944 | 1 | hypothetical protein [Bacillus hypothetical protein BAMF_0901 |
| 1168048 | 1245311 | 77263 |  | orf1221 | 1184941 | 1185903 | 1 | hypothetical protein |
| 1168048 | 1245311 | 77263 | polA | orf1222 | 1186259 | 1189066 | 1 | DNA polymerase I [Bacillus sp. DNA polymerase I |
| 1168048 | 1245311 | 77263 |  | orf1223 | 1189067 | 1189363 | 1 | hypothetical protein [Bacillus hypothetical protein BAMF_0905 |
| 1168048 | 1245311 | 77263 |  | orf1224 | 1189360 | 1190421 | 1 | hypothetical protein |
| 1168048 | 1245311 | 77263 |  | orf1225 | 1190942 | 1191205 | 1 | hypothetical protein |
| 1168048 | 1245311 | 77263 |  | orf1226 | 1191404 | 1191655 | 1 | hypothetical protein |
| 1168048 | 1245311 | 77263 |  | orf1227 | 1191715 | 1192017 | 1 | hypothetical protein |
| 1168048 | 1245311 | 77263 |  | orf1228 | 1192133 | 1192261 | 1 | hypothetical protein |
| 1168048 | 1245311 | 77263 |  | orf1229 | 1192258 | 1192443 | 1 | hypothetical protein |
| 1168048 | 1245311 | 77263 |  | orf1230 | 1192448 | 1192642 | 1 | hypothetical protein |
| 1168048 | 1245311 | 77263 | nrdA | orf1231 | 1193501 | 1195606 | 1 | ribonucleotide-diphosphate reductase subunit alpha |
| 1168048 | 1245311 | 77263 | nrdB | orf1232 | 1195842 | 1196825 | 1 | ribonucleotide-diphosphate reductase |
| 1168048 | 1245311 | 77263 |  | orf1233 | 1196936 | 1197094 | 1 |  |
| 1168048 | 1245311 | 77263 |  | orf1234 | 1197091 | 1197642 | 1 | deoxyuridine 5'-triphosphate nucleotidohydrolase |
| 1168048 | 1245311 | 77263 | thyX | orf1235 | 1197864 | 1198667 | 1 | thymidylate synthase |
| 1168048 | 1245311 | 77263 |  | orf1236 | 1198671 | 1198844 | 1 |  |
| 1168048 | 1245311 | 77263 |  | orf1237 | 1198873 | 1199439 | 1 | SPBc2 prophage-derived protein YorM |
| 1168048 | 1245311 | 77263 |  | orf1238 | 1199639 | 1200658 | 1 | hypothetical protein |
| 1168048 | 1245311 | 77263 |  | orf1239 | 1200655 | 1201230 | 1 | dephospho-CoA kinase |
| 1168048 | 1245311 | 77263 |  | orf1240 | 1201213 | 1201596 | -1 | hypothetical protein |
| 1168048 | 1245311 | 77263 |  | orf1241 | 1201722 | 1202900 | 1 | hypothetical protein |
| 1168048 | 1245311 | 77263 |  | orf1242 | 1202934 | 1203317 | 1 | hypothetical protein |
| 1168048 | 1245311 | 77263 |  | orf1243 | 1203317 | 1203805 | 1 | hypothetical protein |
| 1168048 | 1245311 | 77263 |  | orf1244 | 1203893 | 1204693 | 1 | hypothetical protein |
| 1168048 | 1245311 | 77263 |  | orf1245 | 1205023 | 1205835 | -1 | ATP-dependent DNA ligase |
| 1168048 | 1245311 | 77263 |  | orf1246 | 1205928 | 1206323 | -1 | hypothetical protein [Bacillus hypothetical protein BAMF_0927 |
| 1168048 | 1245311 | 77263 |  | orf1247 | 1206609 | 1207046 | -1 | hypothetical protein |
| 1168048 | 1245311 | 77263 |  | orf1248 | 1207152 | 1207352 | -1 | hypothetical protein [Bacillus hypothetical protein BAMF_0930 |
| 1168048 | 1245311 | 77263 |  | orf1249 | 1207342 | 1207515 | -1 | hypothetical protein [Bacillus hypothetical protein BAMF_0931 |
| 1168048 | 1245311 | 77263 |  | orf1250 | 1207820 | 1208050 | 1 | MULTISPECIES: DNA-binding protein |
| 1168048 | 1245311 | 77263 |  | orf1251 | 1209033 | 1209476 | 1 | hypothetical protein |
| 1168048 | 1245311 | 77263 |  | orf1252 | 1209591 | 1209725 | 1 | hypothetical protein [Bacillus hypothetical protein BAMF_0935 |
| 1168048 | 1245311 | 77263 |  | orf1253 | 1209963 | 1210463 | 1 | hypothetical protein BSNT_10063 |
| 1168048 | 1245311 | 77263 |  | orf1254 | 1210964 | 1211512 | -1 | integrase [Bacillus tyrosine recombinase XerD |
| 1168048 | 1245311 | 77263 |  | orf1255 | 1211595 | 1213349 | 1 | hypothetical protein |
| 1168048 | 1245311 | 77263 |  | orf1256 | 1213579 | 1213797 | 1 | hypothetical protein |
| 1168048 | 1245311 | 77263 |  | orf1257 | 1213800 | 1215431 | 1 | portal protein |
| 1168048 | 1245311 | 77263 |  | orf1258 | 1215431 | 1216255 | 1 | type IV secretion protein Rhs |
| 1168048 | 1245311 | 77263 |  | orf1259 | 1216346 | 1217017 | 1 | Clp protease ClpB |
| 1168048 | 1245311 | 77263 |  | orf1260 | 1217029 | 1218132 | 1 | hypothetical protein |
| 1168048 | 1245311 | 77263 |  | orf1261 | 1218184 | 1218438 | 1 | hypothetical protein [Bacillus hypothetical protein BAMF_0944 |
| 1168048 | 1245311 | 77263 |  | orf1262 | 1218441 | 1218659 | 1 | hypothetical protein [Bacillus hypothetical protein BAMF_0945 |
| 1168048 | 1245311 | 77263 |  | orf1263 | 1218673 | 1219059 | 1 | hypothetical protein [Bacillus hypothetical protein BAMF_0946 |
| 1168048 | 1245311 | 77263 |  | orf1264 | 1219249 | 1219395 | 1 | hypothetical protein [Bacillus hypothetical protein BAXH7_03291 |
| 1168048 | 1245311 | 77263 |  | orf1265 | 1219554 | 1219799 | 1 | hypothetical protein |
| 1168048 | 1245311 | 77263 |  | orf1266 | 1219806 | 1220195 | 1 | hypothetical protein |
| 1168048 | 1245311 | 77263 |  | orf1267 | 1220220 | 1220774 | 1 | hypothetical protein |
| 1168048 | 1245311 | 77263 |  | orf1268 | 1220832 | 1221203 | 1 | hypothetical protein [Bacillus hypothetical protein BAMTA208_16095 |
| 1168048 | 1245311 | 77263 |  | orf1269 | 1221299 | 1221535 | 1 | hypothetical protein [Bacillus hypothetical protein BAMF_0951 |
| 1168048 | 1245311 | 77263 |  | orf1270 | 1221536 | 1224337 | 1 | tail protein |
| 1168048 | 1245311 | 77263 |  | orf1271 | 1224341 | 1225765 | 1 | glycoside hydrolase family 73 |
| 1168048 | 1245311 | 77263 |  | orf1272 | 1225777 | 1227177 | 1 | endopeptidase [Bacillus phage endopeptidase |
| 1168048 | 1245311 | 77263 |  | orf1273 | 1227192 | 1229756 | 1 | peptidase G2 |
| 1168048 | 1245311 | 77263 |  | orf1274 | 1229769 | 1231352 | 1 | hypothetical protein |
| 1168048 | 1245311 | 77263 |  | orf1275 | 1231367 | 1231636 | 1 | hypothetical protein [Bacillus hypothetical protein BAMTA208_16055 |
| 1168048 | 1245311 | 77263 |  | orf1276 | 1231637 | 1231825 | 1 | hypothetical protein [Bacillus hypothetical protein BAMTA208_16050 |
| 1168048 | 1245311 | 77263 |  | orf1277 | 1231955 | 1232116 | -1 |  |
| 1168048 | 1245311 | 77263 | cwlA | orf1278 | 1232115 | 1233026 | 1 | acetylmuramoyl-L-alanine amidase |
| 1168048 | 1245311 | 77263 |  | orf1279 | 1233048 | 1233305 | 1 | MULTISPECIES: holin |
| 1168048 | 1245311 | 77263 |  | orf1280 | 1233398 | 1233958 | 1 | hypothetical protein |
| 1168048 | 1245311 | 77263 |  | orf1281 | 1233974 | 1234309 | -1 | MULTISPECIES: hypothetical protein |
| 1168048 | 1245311 | 77263 |  | orf1282 | 1234355 | 1234567 | -1 | hypothetical protein |
| 1168048 | 1245311 | 77263 |  | orf1283 | 1234727 | 1235512 | 1 | hypothetical protein |
| 1168048 | 1245311 | 77263 |  | orf1284 | 1235954 | 1236310 | -1 | hypothetical protein [Bacillus hypothetical protein BANAU_1128 |
| 1168048 | 1245311 | 77263 |  | orf1285 | 1236544 | 1236675 | 1 | hypothetical protein V529_11420 |
| 1168048 | 1245311 | 77263 |  | orf1286 | 1236676 | 1236975 | -1 | MULTISPECIES: hypothetical protein |
| 1168048 | 1245311 | 77263 |  | orf1287 | 1237141 | 1237335 | -1 | hypothetical protein [Bacillus hypothetical protein BANAU_1131 |
| 1168048 | 1245311 | 77263 |  | orf1288 | 1237369 | 1237761 | -1 | MULTISPECIES: hypothetical protein |
| 1168048 | 1245311 | 77263 |  | orf1289 | 1237773 | 1239551 | -1 | hypothetical protein [Bacillus pyocin S2 |
| 1168048 | 1245311 | 77263 |  | orf1290 | 1240072 | 1240212 | 1 | hypothetical protein [Bacillus hypothetical protein BANAU_1134 |
| 1168048 | 1245311 | 77263 |  | orf1291 | 1240499 | 1241248 | 1 | 2-hydroxy-6-oxononadienedioate/2-hydroxy-6-oxononatrienedioate hydrolase 2 |
| 1168048 | 1245311 | 77263 | hinT | orf1292 | 1241586 | 1241918 | 1 | HIT family hydrolase [Bacillus Histidine triad nucleotide-binding protein 2 |
| 1168048 | 1245311 | 77263 |  | orf1293 | 1241996 | 1242466 | 1 | MULTISPECIES: hypothetical protein |
| 1168048 | 1245311 | 77263 |  | orf1294 | 1243069 | 1243431 | 1 | hypothetical protein [Bacillus hypothetical protein IO97_08015 |
| 1168048 | 1245311 | 77263 |  | orf1295 | 1243793 | 1244077 | 1 | hypothetical protein [Bacillus lactoylglutathione lyase |
| 1168048 | 1245311 | 77263 | penP | orf1296 | 1244397 | 1245311 | 1 | beta-lactamase [Bacillus beta-lactamase |
| 1174058 | 1180056 | 5998 |  | orf1205 | 1173725 | 1174090 | 1 | hypothetical protein |
| 1174058 | 1180056 | 5998 |  | orf1206 | 1174087 | 1174455 | 1 | hypothetical protein |
| 1174058 | 1180056 | 5998 |  | orf1207 | 1174674 | 1175858 | 1 | hypothetical protein |
| 1174058 | 1180056 | 5998 |  | orf1208 | 1175855 | 1176034 | 1 | hypothetical protein |
| 1174058 | 1180056 | 5998 |  | orf1209 | 1176027 | 1176806 | 1 | DNA replication protein |
| 1174058 | 1180056 | 5998 |  | orf1210 | 1176803 | 1177312 | 1 | hypothetical protein |
| 1174058 | 1180056 | 5998 | dnaB | orf1211 | 1177309 | 1178724 | 1 | DNA helicase |
| 1174058 | 1180056 | 5998 |  | orf1212 | 1178859 | 1179854 | 1 | DNA primase |
| 1174058 | 1180056 | 5998 |  | orf1213 | 1179871 | 1180026 | -1 | Xre family transcriptional regulator |
| 1184510 | 1197094 | 12584 |  | orf1220 | 1184510 | 1184944 | 1 | hypothetical protein [Bacillus hypothetical protein BAMF_0901 |
| 1184510 | 1197094 | 12584 |  | orf1221 | 1184941 | 1185903 | 1 | hypothetical protein |
| 1184510 | 1197094 | 12584 | polA | orf1222 | 1186259 | 1189066 | 1 | DNA polymerase I [Bacillus sp. DNA polymerase I |
| 1184510 | 1197094 | 12584 |  | orf1223 | 1189067 | 1189363 | 1 | hypothetical protein [Bacillus hypothetical protein BAMF_0905 |
| 1184510 | 1197094 | 12584 |  | orf1224 | 1189360 | 1190421 | 1 | hypothetical protein |
| 1184510 | 1197094 | 12584 |  | orf1225 | 1190942 | 1191205 | 1 | hypothetical protein |
| 1184510 | 1197094 | 12584 |  | orf1226 | 1191404 | 1191655 | 1 | hypothetical protein |
| 1184510 | 1197094 | 12584 |  | orf1227 | 1191715 | 1192017 | 1 | hypothetical protein |
| 1184510 | 1197094 | 12584 |  | orf1228 | 1192133 | 1192261 | 1 | hypothetical protein |
| 1184510 | 1197094 | 12584 |  | orf1229 | 1192258 | 1192443 | 1 | hypothetical protein |
| 1184510 | 1197094 | 12584 |  | orf1230 | 1192448 | 1192642 | 1 | hypothetical protein |
| 1184510 | 1197094 | 12584 | nrdA | orf1231 | 1193501 | 1195606 | 1 | ribonucleotide-diphosphate reductase subunit alpha |
| 1184510 | 1197094 | 12584 | nrdB | orf1232 | 1195842 | 1196825 | 1 | ribonucleotide-diphosphate reductase |
| 1184510 | 1197094 | 12584 |  | orf1233 | 1196936 | 1197094 | 1 |  |
| 1184510 | 1197094 | 12584 |  | orf1234 | 1197091 | 1197642 | 1 | deoxyuridine 5'-triphosphate nucleotidohydrolase |
| 1210964 | 1217017 | 6053 |  | orf1254 | 1210964 | 1211512 | -1 | integrase [Bacillus tyrosine recombinase XerD |
| 1210964 | 1217017 | 6053 |  | orf1255 | 1211595 | 1213349 | 1 | hypothetical protein |
| 1210964 | 1217017 | 6053 |  | orf1256 | 1213579 | 1213797 | 1 | hypothetical protein |
| 1210964 | 1217017 | 6053 |  | orf1257 | 1213800 | 1215431 | 1 | portal protein |
| 1210964 | 1217017 | 6053 |  | orf1258 | 1215431 | 1216255 | 1 | type IV secretion protein Rhs |
| 1210964 | 1217017 | 6053 |  | orf1259 | 1216346 | 1217017 | 1 | Clp protease ClpB |
| 1764250 | 1769777 | 5527 |  | orf1819 | 1764250 | 1764579 | 1 | integrase [Bacillus prophage LambdaBa04, site-specific recombinase,phage integrase family |
| 1764250 | 1769777 | 5527 |  | orf1820 | 1764994 | 1765713 | 1 | hypothetical protein [Bacillus hypothetical protein BANAU_1663 |
| 1764250 | 1769777 | 5527 |  | orf1821 | 1765901 | 1766185 | -1 | hypothetical protein [Bacillus hypothetical protein BANAU_1664 |
| 1764250 | 1769777 | 5527 |  | orf1822 | 1766191 | 1766709 | -1 | hypothetical protein [Bacillus hypothetical protein BANAU_1665 |
| 1764250 | 1769777 | 5527 |  | orf1823 | 1767783 | 1767932 | 1 | hypothetical protein [Bacillus hypothetical protein MUS_1867 |
| 1764250 | 1769777 | 5527 |  | orf1824 | 1767916 | 1768593 | 1 | polyketide biosynthesis protein |
| 1764250 | 1769777 | 5527 | pksC | orf1825 | 1768908 | 1769777 | 1 | poly(3-hydroxyalkanoate) synthetase |
| 1861041 | 1891050 | 30009 |  | orf1868 | 1861041 | 1861175 | 1 | hypothetical protein [Bacillus hypothetical protein BANAU_1852 |
| 1861041 | 1891050 | 30009 |  | orf1869 | 1862135 | 1862308 | 1 | hypothetical protein, partial |
| 1861041 | 1891050 | 30009 |  | orf1870 | 1862329 | 1862583 | 1 | hypothetical protein, partial |
| 1861041 | 1891050 | 30009 |  | orf1871 | 1863403 | 1863525 | -1 | hypothetical protein O205_00620 |
| 1861041 | 1891050 | 30009 |  | orf1872 | 1863522 | 1864610 | -1 | hypothetical protein [Bacillus putative phage integrase |
| 1861041 | 1891050 | 30009 |  | orf1873 | 1864651 | 1864863 | 1 |  |
| 1861041 | 1891050 | 30009 |  | orf1874 | 1865014 | 1865139 | -1 | hypothetical protein [Bacillus hypothetical protein MUS_2071 |
| 1861041 | 1891050 | 30009 |  | orf1875 | 1865176 | 1866567 | 1 | MFS transporter [Bacillus putative glucitol transport protein gutA |
| 1861041 | 1891050 | 30009 | xynB | orf1876 | 1866796 | 1868211 | 1 | beta-xylosidase [Bacillus xylan 1,4-beta-xylosidase |
| 1861041 | 1891050 | 30009 |  | orf1877 | 1868356 | 1869510 | -1 | xylose repressor [Bacillus Xylose repressor |
| 1861041 | 1891050 | 30009 | xylA | orf1878 | 1869774 | 1871111 | 1 | xylose isomerase [Bacillus xylose isomerase |
| 1861041 | 1891050 | 30009 | xylB | orf1879 | 1871247 | 1872746 | 1 | D-xylulose kinase [Bacillus xylulokinase |
| 1861041 | 1891050 | 30009 | ppsA | orf1880 | 1872921 | 1874525 | 1 | phosphoenolpyruvate synthase |
| 1861041 | 1891050 | 30009 | ppsA | orf1881 | 1874522 | 1875517 | 1 | phosphoenolpyruvate synthase |
| 1861041 | 1891050 | 30009 |  | orf1882 | 1875504 | 1875650 | 1 | hypothetical protein [Bacillus hypothetical protein MUS_2078 |
| 1861041 | 1891050 | 30009 |  | orf1883 | 1876999 | 1877184 | 1 | MULTISPECIES: hypothetical protein |
| 1861041 | 1891050 | 30009 |  | orf1884 | 1877233 | 1877400 | -1 | hypothetical protein [Bacillus hypothetical protein RBAM_017420 |
| 1861041 | 1891050 | 30009 |  | orf1885 | 1877785 | 1878699 | 1 | transcriptional regulator |
| 1861041 | 1891050 | 30009 |  | orf1886 | 1878763 | 1879263 | 1 | MULTISPECIES: DNA damage-inducible protein DinB |
| 1861041 | 1891050 | 30009 | adhP | orf1887 | 1879413 | 1880339 | -1 | acetaldehyde reductase |
| 1861041 | 1891050 | 30009 |  | orf1888 | 1881143 | 1881319 | 1 | hypothetical protein [Bacillus hypothetical protein BANAU_1866 |
| 1861041 | 1891050 | 30009 |  | orf1889 | 1881499 | 1881762 | 1 | hypothetical protein [Bacillus hypothetical protein MUS_2083 |
| 1861041 | 1891050 | 30009 |  | orf1890 | 1882428 | 1883183 | 1 | N-acetyltransferase [Bacillus hypothetical protein BANAU_1868 |
| 1861041 | 1891050 | 30009 |  | orf1891 | 1883512 | 1885311 | 1 | hypothetical protein [Bacillus phage DNA manipulating enzyme |
| 1861041 | 1891050 | 30009 |  | orf1892 | 1885326 | 1885784 | 1 | hypothetical protein [Bacillus putative phage protein YobK |
| 1861041 | 1891050 | 30009 |  | orf1893 | 1885840 | 1886436 | 1 | hypothetical protein [Bacillus hypothetical protein MUS_2092 |
| 1861041 | 1891050 | 30009 |  | orf1894 | 1886532 | 1886891 | 1 | GCN5 family acetyltransferase |
| 1861041 | 1891050 | 30009 |  | orf1895 | 1887206 | 1887544 | 1 | hypothetical protein [Bacillus Prophage-derived-like protein YozM |
| 1861041 | 1891050 | 30009 |  | orf1896 | 1888217 | 1888426 | 1 | hypothetical protein [Bacillus SPBc2 prophage-derived protein YolA |
| 1861041 | 1891050 | 30009 |  | orf1897 | 1889392 | 1889871 | 1 | hypothetical protein [Bacillus hypothetical protein BANAU_1876 |
| 1861041 | 1891050 | 30009 |  | orf1898 | 1889917 | 1890144 | -1 | hypothetical protein IO97_19035 |
| 1861041 | 1891050 | 30009 |  | orf1899 | 1890430 | 1891050 | 1 | hypothetical protein [Bacillus hypothetical protein BANAU_1877 |
| 2172959 | 2229934 | 56975 |  | orf2158 | 2172959 | 2173141 | -1 | MULTISPECIES: regulatory protein |
| 2172959 | 2229934 | 56975 |  | orf2159 | 2173303 | 2173560 | 1 | MULTISPECIES: hypothetical protein |
| 2172959 | 2229934 | 56975 |  | orf2160 | 2173587 | 2173769 | -1 | MULTISPECIES: hypothetical protein |
| 2172959 | 2229934 | 56975 | rnhA | orf2161 | 2173772 | 2174443 | -1 | MULTISPECIES: hypothetical protein |
| 2172959 | 2229934 | 56975 |  | orf2162 | 2174525 | 2175205 | 1 | MULTISPECIES: hypothetical protein |
| 2172959 | 2229934 | 56975 | rnhA | orf2163 | 2175209 | 2175604 | 1 | MULTISPECIES: ribonuclease H RNase H |
| 2172959 | 2229934 | 56975 | sspL | orf2164 | 2175656 | 2175784 | 1 | MULTISPECIES: spore protein SspL |
| 2172959 | 2229934 | 56975 |  | orf2165 | 2175790 | 2176677 | -1 | 5'-3' exonuclease [Bacillus 5'-3' exonuclease |
| 2172959 | 2229934 | 56975 |  | orf2166 | 2176771 | 2176914 | -1 | hypothetical protein [Bacillus hypothetical protein BANAU_2122 |
| 2172959 | 2229934 | 56975 |  | orf2167 | 2176991 | 2177248 | -1 | MULTISPECIES: hypothetical protein |
| 2172959 | 2229934 | 56975 |  | orf2168 | 2177316 | 2180897 | -1 | hypothetical protein [Bacillus hypothetical protein BANAU_2124 |
| 2172959 | 2229934 | 56975 |  | orf2169 | 2180926 | 2181054 | -1 | hypothetical protein [Bacillus hypothetical protein MUS_2381 |
| 2172959 | 2229934 | 56975 | bpsB | orf2170 | 2181248 | 2181754 | -1 | MULTISPECIES: hypothetical protein |
| 2172959 | 2229934 | 56975 | bpsA | orf2171 | 2181754 | 2182854 | -1 | MULTISPECIES: chalcone synthase |
| 2172959 | 2229934 | 56975 |  | orf2172 | 2183021 | 2183629 | 1 | acetyltransferase [Bacillus putative N-acetyltransferase |
| 2172959 | 2229934 | 56975 | ybaZ | orf2173 | 2183658 | 2183966 | -1 | DNA methyltransferase [Bacillus methylated DNA-protein cysteine methyltransferase |
| 2172959 | 2229934 | 56975 |  | orf2174 | 2184189 | 2184452 | 1 | MULTISPECIES: hypothetical protein |
| 2172959 | 2229934 | 56975 |  | orf2175 | 2184926 | 2185042 | 1 | hypothetical protein [Bacillus hypothetical protein MUS_2387 |
| 2172959 | 2229934 | 56975 |  | orf2176 | 2185292 | 2185858 | -1 | isochorismatase [Bacillus isochorismatase |
| 2172959 | 2229934 | 56975 |  | orf2177 | 2186131 | 2186292 | -1 | hypothetical protein [Bacillus hypothetical protein MUS_2389 |
| 2172959 | 2229934 | 56975 |  | orf2178 | 2186476 | 2186667 | -1 | hypothetical protein [Bacillus hypothetical protein MUS_2390 |
| 2172959 | 2229934 | 56975 |  | orf2179 | 2186930 | 2187175 | -1 | DNA-binding protein [Bacillus hypothetical protein BANAU_2132 |
| 2172959 | 2229934 | 56975 |  | orf2180 | 2187690 | 2188382 | 1 | hypothetical protein [Bacillus prophage pi3 protein 59 |
| 2172959 | 2229934 | 56975 | tagE | orf2181 | 2188476 | 2189966 | -1 | poly(glycerol-phosphate)alpha-glucosyltransferase [Bacillus poly(glycerol-phosphate)alpha-glucosyltr |
| 2172959 | 2229934 | 56975 |  | orf2182 | 2190351 | 2190947 | 1 | RNA polymerase subunit sigma-70 |
| 2172959 | 2229934 | 56975 |  | orf2183 | 2191253 | 2191378 | 1 | hypothetical protein [Bacillus hypothetical protein MUS_2396 |
| 2172959 | 2229934 | 56975 |  | orf2184 | 2191878 | 2193041 | -1 | acetylmuramoyl-L-alanine amidase |
| 2172959 | 2229934 | 56975 |  | orf2185 | 2193087 | 2193509 | -1 | holin |
| 2172959 | 2229934 | 56975 |  | orf2186 | 2193559 | 2193744 | -1 | hypothetical protein [Bacillus hypothetical protein BANAU_2138 |
| 2172959 | 2229934 | 56975 |  | orf2187 | 2194103 | 2195926 | -1 | hypothetical protein [Bacillus SPBc2 prophage-derived protein YomR |
| 2172959 | 2229934 | 56975 |  | orf2188 | 2195941 | 2198505 | -1 | peptidase G2 [Bacillus Pre-neck appendage protein Late protein GP12 |
| 2172959 | 2229934 | 56975 |  | orf2189 | 2198559 | 2200262 | -1 | alkaline phosphatase [Bacillus N-acetylmuramoyl-L-alanine amidase |
| 2172959 | 2229934 | 56975 |  | orf2190 | 2200277 | 2201116 | -1 | hypothetical protein [Bacillus hypothetical protein BANAU_2142 |
| 2172959 | 2229934 | 56975 |  | orf2191 | 2201110 | 2205576 | -1 | tail protein [Bacillus SPBc2 prophage-derived transglycosylase |
| 2172959 | 2229934 | 56975 |  | orf2192 | 2205795 | 2206172 | -1 | hypothetical protein [Bacillus hypothetical protein BANAU_2144 |
| 2172959 | 2229934 | 56975 |  | orf2193 | 2206238 | 2206849 | -1 | tail protein [Bacillus putative phagelike major tail protein |
| 2172959 | 2229934 | 56975 |  | orf2194 | 2206861 | 2207244 | -1 | hypothetical protein [Bacillus hypothetical protein BANAU_2146 |
| 2172959 | 2229934 | 56975 |  | orf2195 | 2207241 | 2207639 | -1 | hypothetical protein [Bacillus hypothetical protein BANAU_2147 |
| 2172959 | 2229934 | 56975 |  | orf2196 | 2207636 | 2207953 | -1 | phage head-tail adapter protein |
| 2172959 | 2229934 | 56975 |  | orf2197 | 2207943 | 2208245 | -1 | hypothetical protein [Bacillus hypothetical protein BANAU_2149 |
| 2172959 | 2229934 | 56975 |  | orf2198 | 2208263 | 2208742 | -1 | hypothetical protein [Bacillus Collagen alpha-5(VI) chain Collagen alpha-1(XXIX) chain |
| 2172959 | 2229934 | 56975 |  | orf2199 | 2208765 | 2210057 | -1 | head protein [Bacillus phage major capsid protein, HK97 family |
| 2172959 | 2229934 | 56975 |  | orf2200 | 2210096 | 2210722 | -1 | peptidase U35 [Bacillus Phage head maturation protease |
| 2172959 | 2229934 | 56975 |  | orf2201 | 2210685 | 2211965 | -1 | phage portal protein [Bacillus Portal protein GP3 |
| 2172959 | 2229934 | 56975 |  | orf2202 | 2211970 | 2212140 | -1 | hypothetical protein [Bacillus hypothetical protein BANAU_2154 |
| 2172959 | 2229934 | 56975 |  | orf2203 | 2212154 | 2213863 | -1 | terminase [Bacillus putative terminase large subunit |
| 2172959 | 2229934 | 56975 |  | orf2204 | 2213860 | 2214375 | -1 | terminase [Bacillus Phage terminase, small subunit |
| 2172959 | 2229934 | 56975 |  | orf2205 | 2214339 | 2214485 | -1 | hypothetical protein [Bacillus hypothetical protein MUS_2430 |
| 2172959 | 2229934 | 56975 |  | orf2206 | 2215273 | 2215992 | -1 | hypothetical protein IO97_04255 |
| 2172959 | 2229934 | 56975 |  | orf2207 | 2216015 | 2216827 | -1 | hypothetical protein IO97_04250 |
| 2172959 | 2229934 | 56975 |  | orf2208 | 2217017 | 2217229 | -1 | transcriptional regulator |
| 2172959 | 2229934 | 56975 |  | orf2209 | 2217770 | 2217982 | -1 | transcriptional regulator |
| 2172959 | 2229934 | 56975 |  | orf2210 | 2217990 | 2218127 | -1 | hypothetical protein [Bacillus hypothetical protein IO97_04225 |
| 2172959 | 2229934 | 56975 |  | orf2211 | 2218281 | 2218418 | -1 | hypothetical protein [Bacillus hypothetical protein BACAU_0587 |
| 2172959 | 2229934 | 56975 | rpoE | orf2212 | 2218523 | 2219038 | -1 | positive control sigma-like factor |
| 2172959 | 2229934 | 56975 |  | orf2213 | 2219058 | 2219243 | -1 | hypothetical protein [Bacillus hypothetical protein V529_22710 |
| 2172959 | 2229934 | 56975 |  | orf2214 | 2219491 | 2220156 | -1 | hypothetical protein [Bacillus hypothetical protein IO97_04205 |
| 2172959 | 2229934 | 56975 |  | orf2215 | 2220319 | 2220573 | -1 | hypothetical protein [Bacillus hypothetical protein EF87_21670 |
| 2172959 | 2229934 | 56975 |  | orf2216 | 2220612 | 2220815 | -1 | phage portal protein [Bacillus hypothetical protein V529_22740 |
| 2172959 | 2229934 | 56975 |  | orf2217 | 2220913 | 2221077 | -1 | hypothetical protein V529_22750 |
| 2172959 | 2229934 | 56975 |  | orf2218 | 2221128 | 2221556 | -1 | hypothetical protein [Bacillus hypothetical protein EF87_21685 |
| 2172959 | 2229934 | 56975 |  | orf2219 | 2221652 | 2221795 | -1 | hypothetical protein V529_22770 |
| 2172959 | 2229934 | 56975 |  | orf2220 | 2221792 | 2222532 | -1 | hypothetical protein |
| 2172959 | 2229934 | 56975 |  | orf2221 | 2222624 | 2223325 | -1 | hypothetical protein EF87_20915 |
| 2172959 | 2229934 | 56975 |  | orf2222 | 2223523 | 2224212 | -1 | hypothetical protein EF87_20925 |
| 2172959 | 2229934 | 56975 |  | orf2223 | 2224280 | 2225200 | -1 | hypothetical protein [Bacillus hypothetical protein V529_22820 |
| 2172959 | 2229934 | 56975 |  | orf2224 | 2225197 | 2225385 | -1 | hypothetical protein EF87_20465 |
| 2172959 | 2229934 | 56975 |  | orf2225 | 2225487 | 2225684 | -1 | hypothetical protein [Bacillus skin element |
| 2172959 | 2229934 | 56975 |  | orf2226 | 2225681 | 2225938 | -1 | hypothetical protein [Bacillus hypothetical protein BANAU_2172 |
| 2172959 | 2229934 | 56975 |  | orf2227 | 2225935 | 2226111 | -1 | transcriptional regulator |
| 2172959 | 2229934 | 56975 |  | orf2228 | 2226565 | 2227293 | -1 | kilA [Bacillus Protein kilA |
| 2172959 | 2229934 | 56975 |  | orf2229 | 2227290 | 2227604 | -1 | hypothetical protein V529_22880 |
| 2172959 | 2229934 | 56975 |  | orf2230 | 2227617 | 2227835 | -1 | XRE family transcriptional regulator |
| 2172959 | 2229934 | 56975 |  | orf2231 | 2227989 | 2228366 | 1 | transcriptional regulator |
| 2172959 | 2229934 | 56975 |  | orf2232 | 2228489 | 2228602 | 1 | hypothetical protein [Bacillus hypothetical protein MUS_2454 |
| 2172959 | 2229934 | 56975 |  | orf2233 | 2228738 | 2229934 | 1 | integrase [Bacillus tyrosine recombinase XerC |
| 2767536 | 2778731 | 11195 |  | orf2781 | 2767536 | 2767676 | 1 | hypothetical protein [Bacillus hypothetical protein MUS_3098 |
| 2767536 | 2778731 | 11195 |  | orf2782 | 2767920 | 2768060 | -1 | hypothetical protein [Bacillus hypothetical protein MUS_3099 |
| 2767536 | 2778731 | 11195 |  | orf2783 | 2768038 | 2768388 | 1 | DNA helicase ino80 [Bacillus Transcription elongation factor SPT5 DRB sensitivity-inducing factor la |
| 2767536 | 2778731 | 11195 |  | orf2784 | 2768428 | 2768613 | -1 | MULTISPECIES: hypothetical protein |
| 2767536 | 2778731 | 11195 | hsdS | orf2785 | 2770513 | 2771925 | -1 | type I restriction endonuclease subunit S |
| 2767536 | 2778731 | 11195 | hsdM | orf2786 | 2771922 | 2773352 | -1 | type I restriction endonuclease |
| 2767536 | 2778731 | 11195 | hsdR | orf2787 | 2773485 | 2776640 | -1 | type I restriction endonuclease EcoKI subunit R |
| 2767536 | 2778731 | 11195 |  | orf2788 | 2776975 | 2777151 | -1 | hypothetical protein [Bacillus hypothetical protein MUS_3106 |
| 2767536 | 2778731 | 11195 |  | orf2789 | 2777216 | 2777401 | -1 | integrase/recombinase [Bacillus integrase/recombinase |
| 2767536 | 2778731 | 11195 |  | orf2790 | 2778222 | 2778731 | -1 | metallophosphatase [Bacillus hypothetical protein BANAU_2756 |
| 3453663 | 3476383 | 22720 |  | orf3516 | 3453698 | 3454762 | -1 | MULTISPECIES: saccharopine dehydrogenase |
| 3453663 | 3476383 | 22720 |  | orf3517 | 3454836 | 3456578 | -1 | ABC transporter permease |
| 3453663 | 3476383 | 22720 |  | orf3518 | 3456776 | 3468082 | -1 | non-ribosomal peptide synthetase |
| 3453663 | 3476383 | 22720 |  | orf3519 | 3468094 | 3485196 | -1 | peptide synthetase [Bacillus putative non-ribosomal peptide synthetase |
| 3476400 | 3487625 | 11225 |  | orf3519 | 3468094 | 3485196 | -1 | peptide synthetase [Bacillus putative non-ribosomal peptide synthetase |
| 3476400 | 3487625 | 11225 |  | orf3520 | 3485703 | 3486401 | -1 | 3-oxoacyl-ACP reductase |

**Table S7** Prediction of prophage and CRISPR identification in the ZJU1 genome sequence.

| **#Sequence_Id** | **Sequence_Length** | **CRISPR_Id** | **CRISPR_Description** | **CRISPR_Begin** | **CRISPR_End** | **DR_Length** | **DR_Seq** | **Spacers_Number** | **Spacer_Begin_List** | **Spacer_Length_List** | **Spacer_Seq_List** |
| --- | --- | --- | --- | --- | --- | --- | --- | --- | --- | --- | --- |
| ZJU1 | 4064151 | 1 | Questionable CRISPRs | 706172 | 706362 | 23 | TACGGTTTTGGCGGCTACGGCGG | 3 | 706195 | 19 | CTACCCGGGGTACGGTTTT |
|  |  |  |  |  |  |  |  |  | 706237 | 43 | ATATCCGGGACACGGTTTCGGCGGAGGCTACGGCTACCCGGGA |
|  |  |  |  |  |  |  |  |  | 706303 | 37 | TTTTGGCGGCTACGGCGGCTTCGGGGGATATCCGGGG |

| **Region** | **Region_length** | **Completeness** | **Region_position** | **Possible phage** | **GC_percentage** |
| --- | --- | --- | --- | --- | --- |
| 1 | 77.8Kb | intact | 1165830-1243652 | PHAGE_Paenib_Tripp_NC_028930, ...... | 46.90% |
| 2 | 12Kb | incomplete | 1283055-1295065 | PHAGE_Bacill_SPbeta_NC_001884, ...... | 46.03% |
| 3 | 19.5Kb | incomplete | 1889269-1908801 | PHAGE_Clostr_phiCT453B_NC_029004, ...... | 42.35% |
| 4 | 22.4Kb | incomplete | 2191878-2214375 | PHAGE_Bacill_phi105_NC_004167, ...... | 43.31% |
| 5 | 22.4Kb | incomplete | 2207505-2229934 | PHAGE_Bacill_Stahl_NC_028856, ...... | 40.24% |

**Table S8** Functional classification of KEGG pathway of assembled unigenes.

| **PathWay** | **Pathway_definition** | **number_of_seqs** | **pathway_imagename** |
| --- | --- | --- | --- |
| path:ko01100 | Metabolic pathways | 583 | ko01100.png |
| path:ko01110 | Biosynthesis of secondary metabolites | 277 | ko01110.png |
| path:ko01120 | Microbial metabolism in diverse environments | 158 | ko01120.png |
| path:ko01230 | Biosynthesis of amino acids | 121 | ko01230.png |
| path:ko02010 | ABC transporters | 120 | ko02010.png |
| path:ko02020 | Two-component system | 97 | ko02020.png |
| path:ko01200 | Carbon metabolism | 96 | ko01200.png |
| path:ko00230 | Purine metabolism | 65 | ko00230.png |
| path:ko00240 | Pyrimidine metabolism | 55 | ko00240.png |
| path:ko00620 | Pyruvate metabolism | 44 | ko00620.png |
| path:ko00330 | Arginine and proline metabolism | 42 | ko00330.png |
| path:ko03010 | Ribosome | 41 | ko03010.png |
| path:ko00520 | Amino sugar and nucleotide sugar metabolism | 40 | ko00520.png |
| path:ko00010 | Glycolysis / Gluconeogenesis | 40 | ko00010.png |
| path:ko00270 | Cysteine and methionine metabolism | 37 | ko00270.png |
| path:ko00190 | Oxidative phosphorylation | 35 | ko00190.png |
| path:ko00250 | Alanine, aspartate and glutamate metabolism | 34 | ko00250.png |
| path:ko02040 | Flagellar assembly | 33 | ko02040.png |
| path:ko00260 | Glycine, serine and threonine metabolism | 32 | ko00260.png |
| path:ko01212 | Fatty acid metabolism | 32 | ko01212.png |
| path:ko01210 | 2-Oxocarboxylic acid metabolism | 31 | ko01210.png |
| path:ko00720 | Carbon fixation pathways in prokaryotes | 31 | ko00720.png |
| path:ko00500 | Starch and sucrose metabolism | 30 | ko00500.png |
| path:ko00970 | Aminoacyl-tRNA biosynthesis | 27 | ko00970.png |
| path:ko00640 | Propanoate metabolism | 26 | ko00640.png |
| path:ko00680 | Methane metabolism | 26 | ko00680.png |
| path:ko00030 | Pentose phosphate pathway | 25 | ko00030.png |
| path:ko00061 | Fatty acid biosynthesis | 25 | ko00061.png |
| path:ko00020 | Citrate cycle (TCA cycle) | 25 | ko00020.png |
| path:ko00630 | Glyoxylate and dicarboxylate metabolism | 25 | ko00630.png |
| path:ko00280 | Valine, leucine and isoleucine degradation | 24 | ko00280.png |
| path:ko00400 | Phenylalanine, tyrosine and tryptophan biosynthesis | 23 | ko00400.png |
| path:ko00650 | Butanoate metabolism | 22 | ko00650.png |
| path:ko02030 | Bacterial chemotaxis | 22 | ko02030.png |
| path:ko03430 | Mismatch repair | 21 | ko03430.png |
| path:ko03440 | Homologous recombination | 21 | ko03440.png |
| path:ko02060 | Phosphotransferase system (PTS) | 21 | ko02060.png |
| path:ko00770 | Pantothenate and CoA biosynthesis | 21 | ko00770.png |
| path:ko00564 | Glycerophospholipid metabolism | 21 | ko00564.png |
| path:ko00051 | Fructose and mannose metabolism | 20 | ko00051.png |
| path:ko00040 | Pentose and glucuronate interconversions | 20 | ko00040.png |
| path:ko00790 | Folate biosynthesis | 20 | ko00790.png |
| path:ko00052 | Galactose metabolism | 20 | ko00052.png |
| path:ko03060 | Protein export | 19 | ko03060.png |
| path:ko03030 | DNA replication | 19 | ko03030.png |
| path:ko00860 | Porphyrin and chlorophyll metabolism | 19 | ko00860.png |
| path:ko00780 | Biotin metabolism | 19 | ko00780.png |
| path:ko00340 | Histidine metabolism | 18 | ko00340.png |
| path:ko00910 | Nitrogen metabolism | 18 | ko00910.png |
| path:ko00300 | Lysine biosynthesis | 17 | ko00300.png |
| path:ko00071 | Fatty acid degradation | 16 | ko00071.png |
| path:ko00550 | Peptidoglycan biosynthesis | 16 | ko00550.png |
| path:ko00730 | Thiamine metabolism | 16 | ko00730.png |
| path:ko03410 | Base excision repair | 15 | ko03410.png |
| path:ko00561 | Glycerolipid metabolism | 15 | ko00561.png |
| path:ko00920 | Sulfur metabolism | 15 | ko00920.png |
| path:ko04112 | Cell cycle - Caulobacter | 14 | ko04112.png |
| path:ko00900 | Terpenoid backbone biosynthesis | 14 | ko00900.png |
| path:ko03070 | Bacterial secretion system | 14 | ko03070.png |
| path:ko00562 | Inositol phosphate metabolism | 14 | ko00562.png |
| path:ko00310 | Lysine degradation | 13 | ko00310.png |
| path:ko00670 | One carbon pool by folate | 13 | ko00670.png |
| path:ko00290 | Valine, leucine and isoleucine biosynthesis | 13 | ko00290.png |
| path:ko00760 | Nicotinate and nicotinamide metabolism | 12 | ko00760.png |
| path:ko00380 | Tryptophan metabolism | 12 | ko00380.png |
| path:ko00710 | Carbon fixation in photosynthetic organisms | 12 | ko00710.png |
| path:ko03018 | RNA degradation | 12 | ko03018.png |
| path:ko03420 | Nucleotide excision repair | 12 | ko03420.png |
| path:ko04122 | Sulfur relay system | 12 | ko04122.png |
| path:ko01054 | Nonribosomal peptide structures | 11 | ko01054.png |
| path:ko00130 | Ubiquinone and other terpenoid-quinone biosynthesis | 11 | ko00130.png |
| path:ko00450 | Selenocompound metabolism | 11 | ko00450.png |
| path:ko00410 | beta-Alanine metabolism | 10 | ko00410.png |
| path:ko00312 | beta-Lactam resistance | 10 | ko00312.png |
| path:ko04146 | Peroxisome | 10 | ko04146.png |
| path:ko00350 | Tyrosine metabolism | 10 | ko00350.png |
| path:ko00660 | C5-Branched dibasic acid metabolism | 9 | ko00660.png |
| path:ko01040 | Biosynthesis of unsaturated fatty acids | 9 | ko01040.png |
| path:ko00627 | Aminobenzoate degradation | 9 | ko00627.png |
| path:ko00521 | Streptomycin biosynthesis | 9 | ko00521.png |
| path:ko00195 | Photosynthesis | 8 | ko00195.png |
| path:ko00480 | Glutathione metabolism | 8 | ko00480.png |
| path:ko00625 | Chloroalkane and chloroalkene degradation | 8 | ko00625.png |
| path:ko00360 | Phenylalanine metabolism | 8 | ko00360.png |
| path:ko00072 | Synthesis and degradation of ketone bodies | 7 | ko00072.png |
| path:ko00430 | Taurine and hypotaurine metabolism | 7 | ko00430.png |
| path:ko05150 | Staphylococcus aureus infection | 7 | ko05150.png |
| path:ko04066 | HIF-1 signaling pathway | 7 | ko04066.png |
| path:ko00473 | D-Alanine metabolism | 6 | ko00473.png |
| path:ko00362 | Benzoate degradation | 6 | ko00362.png |
| path:ko00983 | Drug metabolism - other enzymes | 6 | ko00983.png |
| path:ko00053 | Ascorbate and aldarate metabolism | 6 | ko00053.png |
| path:ko00903 | Limonene and pinene degradation | 6 | ko00903.png |
| path:ko00253 | Tetracycline biosynthesis | 6 | ko00253.png |
| path:ko01053 | Biosynthesis of siderophore group nonribosomal peptides | 6 | ko01053.png |
| path:ko03020 | RNA polymerase | 5 | ko03020.png |
| path:ko04068 | FoxO signaling pathway | 5 | ko04068.png |
| path:ko00750 | Vitamin B6 metabolism | 5 | ko00750.png |
| path:ko00471 | D-Glutamine and D-glutamate metabolism | 5 | ko00471.png |
| path:ko00740 | Riboflavin metabolism | 5 | ko00740.png |
| path:ko00401 | Novobiocin biosynthesis | 5 | ko00401.png |
| path:ko00460 | Cyanoamino acid metabolism | 5 | ko00460.png |
| path:ko01220 | Degradation of aromatic compounds | 5 | ko01220.png |
| path:ko04070 | Phosphatidylinositol signaling system | 4 | ko04070.png |
| path:ko00523 | Polyketide sugar unit biosynthesis | 4 | ko00523.png |
| path:ko05132 | Salmonella infection | 4 | ko05132.png |
| path:ko05014 | Amyotrophic lateral sclerosis (ALS) | 4 | ko05014.png |
| path:ko04626 | Plant-pathogen interaction | 4 | ko04626.png |
| path:ko00960 | Tropane, piperidine and pyridine alkaloid biosynthesis | 4 | ko00960.png |
| path:ko05134 | Legionellosis | 4 | ko05134.png |
| path:ko00830 | Retinol metabolism | 3 | ko00830.png |
| path:ko05152 | Tuberculosis | 3 | ko05152.png |
| path:ko00590 | Arachidonic acid metabolism | 3 | ko00590.png |
| path:ko00950 | Isoquinoline alkaloid biosynthesis | 3 | ko00950.png |
| path:ko00791 | Atrazine degradation | 3 | ko00791.png |
| path:ko00626 | Naphthalene degradation | 3 | ko00626.png |
| path:ko00982 | Drug metabolism - cytochrome P450 | 3 | ko00982.png |
| path:ko03450 | Non-homologous end-joining | 3 | ko03450.png |
| path:ko00311 | Penicillin and cephalosporin biosynthesis | 3 | ko00311.png |
| path:ko05120 | Epithelial cell signaling in Helicobacter pylori infection | 3 | ko05120.png |
| path:ko04141 | Protein processing in endoplasmic reticulum | 3 | ko04141.png |
| path:ko04727 | GABAergic synapse | 3 | ko04727.png |
| path:ko04724 | Glutamatergic synapse | 3 | ko04724.png |
| path:ko00785 | Lipoic acid metabolism | 3 | ko00785.png |
| path:ko05111 | Vibrio cholerae pathogenic cycle | 3 | ko05111.png |
| path:ko05016 | Huntington's disease | 3 | ko05016.png |
| path:ko03320 | PPAR signaling pathway | 3 | ko03320.png |
| path:ko00980 | Metabolism of xenobiotics by cytochrome P450 | 3 | ko00980.png |
| path:ko04011 | MAPK signaling pathway - yeast | 2 | ko04011.png |
| path:ko04920 | Adipocytokine signaling pathway | 2 | ko04920.png |
| path:ko03013 | RNA transport | 2 | ko03013.png |
| path:ko00600 | Sphingolipid metabolism | 2 | ko00600.png |
| path:ko05206 | MicroRNAs in cancer | 2 | ko05206.png |
| path:ko04964 | Proximal tubule bicarbonate reclamation | 2 | ko04964.png |
| path:ko00642 | Ethylbenzene degradation | 2 | ko00642.png |
| path:ko00440 | Phosphonate and phosphinate metabolism | 2 | ko00440.png |
| path:ko05010 | Alzheimer's disease | 2 | ko05010.png |
| path:ko04917 | Prolactin signaling pathway | 2 | ko04917.png |
| path:ko00511 | Other glycan degradation | 2 | ko00511.png |
| path:ko00281 | Geraniol degradation | 2 | ko00281.png |
| path:ko05204 | Chemical carcinogenesis | 2 | ko05204.png |
| path:ko05200 | Pathways in cancer | 2 | ko05200.png |
| path:ko00945 | Stilbenoid, diarylheptanoid and gingerol biosynthesis | 1 | ko00945.png |
| path:ko05203 | Viral carcinogenesis | 1 | ko05203.png |
| path:ko05020 | Prion diseases | 1 | ko05020.png |
| path:ko00624 | Polycyclic aromatic hydrocarbon degradation | 1 | ko00624.png |
| path:ko04621 | NOD-like receptor signaling pathway | 1 | ko04621.png |
| path:ko00472 | D-Arginine and D-ornithine metabolism | 1 | ko00472.png |
| path:ko05215 | Prostate cancer | 1 | ko05215.png |
| path:ko04973 | Carbohydrate digestion and absorption | 1 | ko04973.png |
| path:ko04612 | Antigen processing and presentation | 1 | ko04612.png |
| path:ko00633 | Nitrotoluene degradation | 1 | ko00633.png |
| path:ko04940 | Type I diabetes mellitus | 1 | ko04940.png |
| path:ko04918 | Thyroid hormone synthesis | 1 | ko04918.png |
| path:ko01055 | Biosynthesis of vancomycin group antibiotics | 1 | ko01055.png |
| path:ko04914 | Progesterone-mediated oocyte maturation | 1 | ko04914.png |
| path:ko04910 | Insulin signaling pathway | 1 | ko04910.png |
| path:ko01051 | Biosynthesis of ansamycins | 1 | ko01051.png |
| path:ko00603 | Glycosphingolipid biosynthesis - globo series | 1 | ko00603.png |
| path:ko04142 | Lysosome | 1 | ko04142.png |
| path:ko00510 | N-Glycan biosynthesis | 1 | ko00510.png |
| path:ko04930 | Type II diabetes mellitus | 1 | ko04930.png |
| path:ko03008 | Ribosome biogenesis in eukaryotes | 1 | ko03008.png |
| path:ko00622 | Xylene degradation | 1 | ko00622.png |
| path:ko00908 | Zeatin biosynthesis | 1 | ko00908.png |
| path:ko04915 | Estrogen signaling pathway | 1 | ko04915.png |
| path:ko00121 | Secondary bile acid biosynthesis | 1 | ko00121.png |
| path:ko00524 | Butirosin and neomycin biosynthesis | 1 | ko00524.png |
| path:ko00906 | Carotenoid biosynthesis | 1 | ko00906.png |
| path:ko05340 | Primary immunodeficiency | 1 | ko05340.png |
| path:ko00361 | Chlorocyclohexane and chlorobenzene degradation | 1 | ko00361.png |
| path:ko05146 | Amoebiasis | 1 | ko05146.png |
| path:ko05205 | Proteoglycans in cancer | 1 | ko05205.png |
| path:ko04152 | AMPK signaling pathway | 1 | ko04152.png |
| path:ko05211 | Renal cell carcinoma | 1 | ko05211.png |
| path:ko00621 | Dioxin degradation | 1 | ko00621.png |
| path:ko04151 | PI3K-Akt signaling pathway | 1 | ko04151.png |
| path:ko04978 | Mineral absorption | 1 | ko04978.png |
| path:ko00363 | Bisphenol degradation | 1 | ko00363.png |
| path:ko00120 | Primary bile acid biosynthesis | 1 | ko00120.png |
| path:ko00592 | alpha-Linolenic acid metabolism | 1 | ko00592.png |

**Table S9** *Bacillus* spp. used for comparative genomic analysis

| **Accession** | **Organism** |
| --- | --- |
| CP010997 | *Bacillus pumilus* SH-B11 |
| CP018574 | *Bacillus pumilus* GLB197 |
| AP014928 | *Bacillus pumilus* TUAT1 |
| CP022477 | *Bacillus licheniformis* BL-010 |
| CP023729 | *Bacillus licheniformis* ATCC 9789 |
| CP006952 | *Bacillus amyloliquefaciens* LFB112 |
| CP020893 | *Bacillus vallismortis* NBIF-001 |
| CP000560 | *Bacillus amyloliquefaciens* FZB42^T^ |
| CP021890 | *Bacillus velezensis* SRCM101413 |
| AJVF01 | *Bacillus siamensis* KCTC 13613^T^ |
| CP002634 | *Bacillus amyloliquefaciens* LL3 |
| CP004405 | *Bacillus subtilis* subsp. *subtilis* BAB-1 |
| CP002468 | *Bacillus subtilis* BSn5 |
| AP011541 | *Bacillus subtilis* subsp. *natto* BEST195 |
| CP003695 | *Bacillus subtilis* subsp. *subtilis* strain BSP1 |
| CP002905 | *Bacillus subtilis* subsp. *spizizenii* TU-B-10^T^ |
| LDYG01 | *Bacillus coahuilensis* p1.1.43 |
| NC002570.2 | *Bacillus halodurans* C-125 |
| AP006627 | *Bacillus clausii* KSM-K16 |
| CP000764 | *Bacillus cytotoxicus* NVH 391-98 |
| CM000745 | *Bacillus pseudomycoides* DSM 12442 |
| CP041691 | *Bacillus amyloliquefaciens* ZJU1 |
| CP009692 | *Bacillus mycoides* ATCC 6462 |
| AE017355 | *Bacillus thuringiensis* serovar konkukian str. 97-27 |
| AE016879 | *Bacillus anthracis* str. Ames |
| AE016877 | *Bacillus cereus* ATCC 14579^T^ |
| CP000002 | *Bacillus licheniformis* DSM13^T^ |
| FN597644 | *Bacillus amyloliquefaciens* DSM7^T^ |
| AL009126 | *Bacillus subtilis* subsp. *subtilis* 168^T^ (DSM10^T^) |
| CP022653 | *Bacillus atrophaeus* GQJK17 |
| CP000813 | *Bacillus pumilus* SAFR-032 |
| AMSH01 | *Bacillus xiamenensis* HYC-10^T^ |
| LLZC01 | *Bacillus velezensis NRRL* B-41580^T^ |
| JPYY01 | *Bacillus subtilis* NKYL29 |
| HG514499 | *Bacillus amyloliquefaciens* subsp*. plantarum* NAU-B3 |
| CP021890 | *Bacillus velezensis* SRCM101413 |
| CP013727 | *Bacillus amyloliquefaciens* MBE1283 |
| CP009679 | *Bacillus methylotrophicus* JS25R |
| CP003332 | *Bacillus amyloliquefaciens* Y2 |
| AVQH01 | *Bacillus amyloliquefaciens* EGD-AQ14 |
| AQGM01 | *Bacillus subtilis* SPZ1 |
| AOFO01 | *Bacillus methylotrophicus* SK19.001 |

**Table S10** Identification of gene clusters potentially involved in antimicrobial metabolite synthesis in *B. amyloliquefaciens* ZJU1.

| **Type** | **From** | **To** | **Most similar known cluster** | **MIBiG BGC-ID** |
| --- | --- | --- | --- | --- |
| Nrps | 314489 | 379896 | Surfactin_biosynthetic_gene_cluster (82% of genes show similarity) | BGC0000433_c1 |
| Cf_saccharide | 718721 | 748313 | Plantathiazolicin_/_plantazolicin_biosynthetic_gene_cluster (41% of genes show similarity) | BGC0000569_c1 |
| Other_ks | 942322 | 983566 | Butirosin_biosynthetic_gene_cluster (7% of genes show similarity) | BGC0000693_c1 |
| Cf_fatty_acid | 1100481 | 1125275 | Citrulline_biosynthetic_gene_cluster (27% of genes show similarity) | BGC0000895_c1 |
| Transatpks | 1441990 | 1527892 | Macrolactin_biosynthetic_gene_cluster (100% of genes show similarity) | BGC0000181_c1 |
| Transatpks-Nrps | 1756327 | 1859007 | Bacillaene_biosynthetic_gene_cluster (100% of genes show similarity) | BGC0001089_c1 |
| Transatpks-Nrps | 1925850 | 2063680 | Fengycin_biosynthetic_gene_cluster (100% of genes show similarity) | BGC0001095_c1 |
| Transatpks | 2361210 | 2461654 | Difficidin_biosynthetic_gene_cluster (100% of genes show similarity) | BGC0000176_c1 |
| Bacteriocin-Nrps | 3103866 | 3170662 | Bacillibactin_biosynthetic_gene_cluster (100% of genes show similarity) | BGC0000309_c1 |
| Cf_saccharide | 3508675 | 3563486 | Teichuronic_acid_biosynthetic_gene_cluster (100% of genes show similarity) | BGC0000868_c1 |
| Cf_other | 3707421 | 3779860 | Bacilysin_biosynthetic_gene_cluster (100% of genes show similarity) | BGC0001184_c1 |
| Lantipeptide | 3903102 | 3927086 | Mersacidin_biosynthetic_gene_cluster (100% of genes show similarity) | BGC0000527_c1 |
| Cf_putative | 389171 | 406345 | - | - |
| Cf_fatty_acid | 560109 | 585166 | - | - |
| Phosphonate | 625719 | 666603 | - | - |
| Cf_putative | 829660 | 850824 | - | - |
| Cf_fatty_acid | 1011629 | 1032606 | - | - |
| Cf_putative | 1034529 | 1042289 | - | - |
| Terpene | 1065881 | 1086621 | - | - |
| Cf_putative | 1146222 | 1155133 | - | - |
| Cf_putative | 1261815 | 1278423 | - | - |
| Terpene | 2089507 | 2111390 | - | - |
| T3pks-Cf_saccharide | 2151344 | 2209966 | - | - |
| Cf_putative | 2657439 | 2665903 | - | - |
| Cf_putative | 3064464 | 3077296 | - | - |
| Cf_saccharide | 3353785 | 3379196 | - | - |
| Nrps | 3436776 | 3505196 | - | - |
| Cf_putative | 3590761 | 3607149 | - | - |
| Cf_putative | 3931344 | 3945034 | - | - |
| Cf_saccharide | 3995337 | 4020211 | - | - |
| Cf_putative | 4028649 | 4044066 | - | - |
